# Detailed Anatomical and Electrophysiological Models of Human Atria and Torso for the Simulation of Atrial Activation

**DOI:** 10.1371/journal.pone.0141573

**Published:** 2015-11-02

**Authors:** Ana Ferrer, Rafael Sebastián, Damián Sánchez-Quintana, José F. Rodríguez, Eduardo J. Godoy, Laura Martínez, Javier Saiz

**Affiliations:** 1 Centro de Investigación e Innovación en Bioingeniería (Ci2B), Universitat Politècnica de València, Valencia, Spain; 2 Computational Multiscale Physiology Lab (CoMMLab), Department of Computer Science, Universitat de Valencia, Valencia, Spain; 3 Department of Anatomy and Cell Biology, Faculty of Medicine, Universidad de Extremadura, Badajoz, Spain; 4 Applied Mechanics and Bioengineering Group (AMB), Universidad de Zaragoza, Zaragoza, Spain, and Dipartimento di Chimica, Materiali e Ingegneria Chimica “Giulio Natta”, Politecnico di Milano, Milano, Italy; Gent University, BELGIUM

## Abstract

Atrial arrhythmias, and specifically atrial fibrillation (AF), induce rapid and irregular activation patterns that appear on the torso surface as abnormal P-waves in electrocardiograms and body surface potential maps (BSPM). In recent years both P-waves and the BSPM have been used to identify the mechanisms underlying AF, such as localizing ectopic foci or high-frequency rotors. However, the relationship between the activation of the different areas of the atria and the characteristics of the BSPM and P-wave signals are still far from being completely understood. In this work we developed a multi-scale framework, which combines a highly-detailed 3D atrial model and a torso model to study the relationship between atrial activation and surface signals in sinus rhythm. Using this multi scale model, it was revealed that the best places for recording P-waves are the frontal upper right and the frontal and rear left quadrants of the torso. Our results also suggest that only nine regions (of the twenty-one structures in which the atrial surface was divided) make a significant contribution to the BSPM and determine the main P-wave characteristics.

## Introduction

Atrial arrhythmias, mainly atrial fibrillation (AF), are the most common sustained cardiac arrhythmias in humans and are considered major causes of morbidity and mortality [1]. In recent decades this has led to the in-depth study of the human atrial anatomy and electrophysiology from cellular to tissue scale, which has provided a large amount of information on the atrial structure and function. However collecting all this multi-scale heterogeneous information in order to understand pathological mechanisms is somewhat complex. Atrial arrhythmias induce rapid and irregular activation patterns that are reflected as irregular P-wave morphology in the electrocardiogram (ECG) and as irregular atrial activity in body surface potential maps (BSPM) recorded from multi-electrodes [2]. However, the relationship between the atrial electrophysiological function, the electrocardiogram (ECG), which is still the primary source of information for diagnosis, and the BSPM still presents many challenges and unknowns that cannot be solved experimentally. Although recent studies have used the electrocardiographic P-wave and the BSPM characteristics to localize high-frequency rotors on the atrial surface in AF [2] or to detect atrial structural abnormalities [3], all of them highlight the difficulty of separating the contribution of the different atrial structures to P-waves and the BSPM.

Computational modelling provides a framework for integrating multi-scale models to solve detailed atrial electrophysiology together with the forward problem in cardiology in order to link atrial activity with the recorded signals, such as electrograms (EGM), ECG and the BSPM [4]. Such a framework has to include all the available multi-scale information, from sub-cellular processes up to the body scale, and detailed descriptions of atrial and body anatomy, to provide meaningful and realistic results. For instance, it is well known that the microstructure of the atrial tissue plays a key role in the sequence of electrical activation, in both healthy and diseased subjects [5]. The sequence of activation and the direction of the depolarisation wavefront are strongly dependent on the preferential conduction bundles and the myofibre architecture [6]. As regards electrophysiology, it is known that the atrial cells and tissue have heterogeneous properties, which give rise to different action potential morphologies. Since not all the necessary functional and anatomical data required for the models is available, it is usually complemented with data collected from different animals or human ex-vivo histological samples collected ad-hoc to overcome these limitations.

Several computational models of the atria have already incorporated important features, such as detailed myofibre orientation and specialised fast conduction pathways from histological studies [7,8], from micro-CT studies [9] or from diffusion tensor magnetic resonance imaging (DT-MRI) [10]. However, these models are usually built from different animal data or are based on incomplete histological descriptions from the literature. Moreover, most of the models do not take the torso into account and therefore cannot be used to obtain the ECG or to study the relationship between internal and external electrical signals.

The main objective of this work is to present a complete three-dimensional model of the human atria and torso to elucidate the relationship between internal electrical signals and body surface maps, by identifying the contribution of the electrical sources from each region of the atria on the surface signals. To obtain reliable results, our previously developed atrial model [8] was improved anatomically with new histological information. Cellular and tissue heterogeneities were also redefined using the cellular Maleckar model [11] and considering the experimental results available. Finally, we also developed a torso model that included the geometrical and electrical properties of the principal organs. We used this integrated model to perform multi-scale electrophysiology simulations and to analyse the contribution of different areas of the atria to the different phases of the P-wave from the cellular level up to the torso scale. We also analysed the body surface patterns to elucidate the spatial and temporal contribution of each atrial region to the total surface potential map and to show the best regions of the torso for detecting the activation of the different atrial structures.

## Material and Methods

### A. Analysis of histological data

The anatomical data used in this study has been gathered over recent years. In particular 30 formalin-fixed hearts from patients who died of non-cardiac causes has been considered [12–15]. This study has been approved by the bioethical committee on human research (University of Extremadura, registration n°14/2015). The mean heart weight was 375±25 g (range 325–420 g). The hearts selected to represent the spectrum of morphological features were dissected and prepared for light microscopy to perform the histological studies. Blocks of tissue encompassing various atrial regions were processed and serially sectioned at 12 or 15 μm in sagittal or anteroposterior, frontal and transverse planes. Sections were stained at 1 mm intervals with Masson’s trichrome [12]. Photographs were taken using a stereo microscope equipped with a digital camera (Nikon SM 1500). The resolution of the histological images was 300 pixels/inch (width: 2560 pixels, height 1920 pixels). The microscope software was used to perform different measurements.

The alignment of the myocardial fibres of the atria was studied by peeling away the epicardium and the endocardium to reveal the arrangement of the major muscular bundles that make up the walls of the atrial chambers. The macroscopic appearance of slender muscle bundles revealed three main orientations: circumferential, longitudinal, and oblique. Circumferential fibres were those parallel to the insertion of the tricuspid and mitral valves; longitudinal fibres were perpendicular to the circumferential fibres, and oblique fibres were at angles to both.

**Fig 1:**
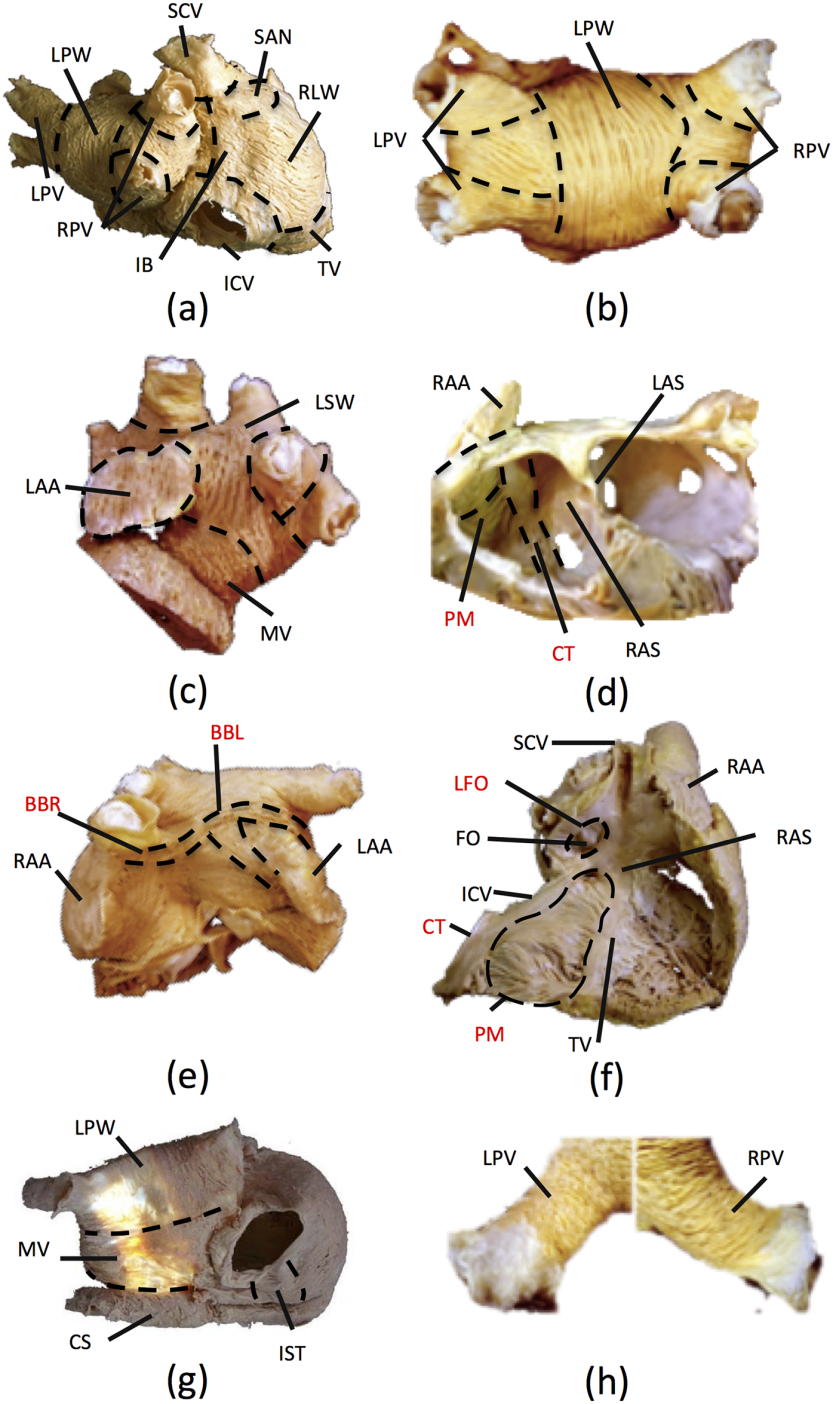
Anatomical atrial regions and principal fibre direction. a)-h) Regions are delimited by dashed lines, displaying different atrial views with circumferential, longitudinal and oblique fibre directions; The preferential conduction axis are labelled in red colour. Panels b), c), d) and e) are adapted from [13–15] under a CC BY license, with permission from Ho SY and Sánchez-Quintana D, original copyright years 2012 and 2013.

A total of 21 anatomical atrial regions were identified, and the principal direction of the fibres within each region was histologically analysed (Fig 1a–1h). With the exception of views (b) and (h) which were common to 60% of the hearts studied, the fibre orientations shown in all the other views were observed in 80% of the hearts. In the right atria (RA), 12 regions were recognised: sinoatrial node (SAN), Crista Terminalis (CT), Bachmann bundle (right part, BBR), intercaval bundle (IB), septum (RAS), lateral wall (RLW), right appendage (RAA), pectinate muscles (PM), isthmus (IST), superior (SCV) and inferior cava veins (ICV) and ring of the tricuspid valve (TV). In the left atria (LA) 8 regions were identified besides the left section of the Bachmann bundle (BBL): superior wall (LSW), septum (LAS), left appendage (LAA), posterior wall (LPW), ring of the mitral valve (MV), right (RPV) and left pulmonary veins (LPV) and the coronary sinus (CS). In addition, the fossa ovalis (FO) and its limb (LFO), which connects the RA and the LA, were considered as one independent structure.

After anatomically defining the boundaries of the regions, the principal fibre orientation was determined with respect to the global coordinate system of the atria by means of a vector in 3D. All vectors were considered tangential to the epicardial wall plane. Some structures, such as the SCV and the ICV, the TV and the MV, the RPV and the LPV, and the CS showed a circumferential fibre orientation. RA structures like the IB, the RLW and the RAA have homogeneously oriented fibres forming an approximate angle of 60° with respect to the CT, whereas RAS fibres are almost parallel to the CT. On the other hand, the IST fibres are parallel to those of the ICV and the TV. In the case of the LA, the fibres from the LAS are aligned parallel to their adjacent regions in the RAS (actually there is only one septum with two faces separated by an insulating layer). Fibres from the LPW descend from the LSW perpendicularly to the fibres in the junction between the RPVs and the LPVs with the MV. Finally, the LAA, the most prominent structure in the LA, aligns its fibres perpendicularly to those in the LSW.

Special emphasis was made on determining the morphology and location of the main conduction bundles. We observed three main structures: the CT, the PMs and the BB (Fig 1d–1f, red labels). The CT runs transmurally from the base of the SCV to the ICV with an average thickness of 3mm (Fig 1d and 1f). The PMs were identified as tubular structures that run on the luminal surface, almost parallel to each other, with a thickness of 1–2 mm. A total of 15 to 34 PMs were observed to be distributed along the CT and extended towards the vestibule (Fig 1d), in agreement with previous studies [16]. The BB was identified as a longitudinal two-sided structure connecting both atria. Its right side (BBR) starts at the SAN and embraces the SCV towards the LA. In the left side (BBL), the BB is divided into two main axes that extend around the LAA from both sides (Fig 1e).

### B. 3D Model of the human atria

**Fig 2:**
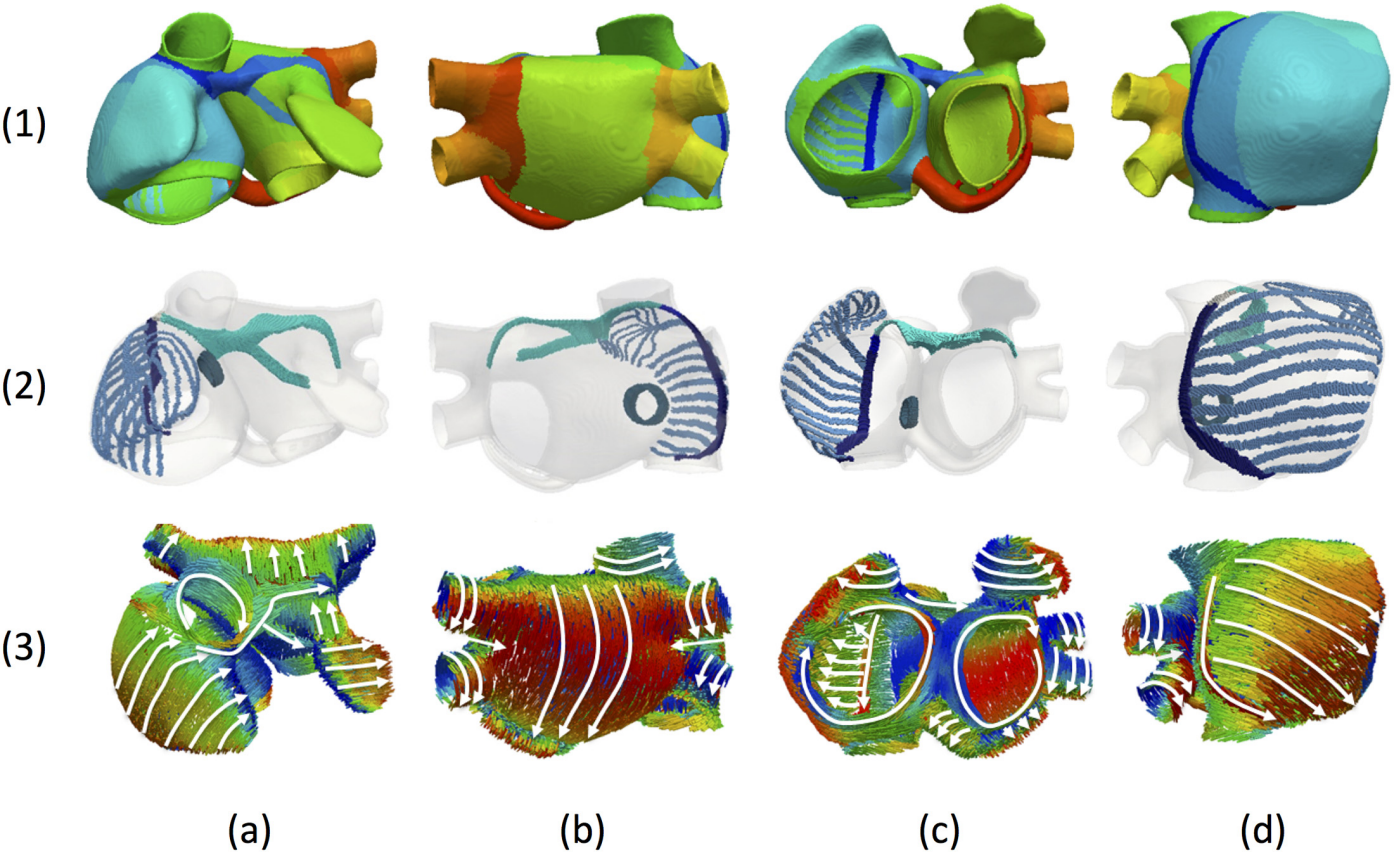
Properties of the 3D atrial model. Row (1) show in colours the division in 21 atrial regions; Row (2) shows preferential conduction bundles; Row (3) shows principal fibre direction; Columns correspond to a) Frontal view; b) rear view; c) inferior view; and d) right lateral view.

In the present work, we developed a new atrial model that improves our previous 3D model of the human atria [8]. The new model (RIUNET, http://hdl.handle.net/10251/55150) has enhanced anatomical and functional heterogeneity and the detailed regional description of fibre direction. The resulting computational finite element model is a multi-layer mesh with a homogeneous wall thickness between 600 and 900 μm (754.893 nodes and 515.010 elements), built with linear hexahedral elements with regular spatial resolution of 300 μm. Following the histological analysis, the model was manually divided into 21 regions. However, for the detailed description of fibre orientation, some regions were further subdivided into a total of 53 sub-regions (Fig 2 row 1, and S1 Table).

The model also includes the preferential conduction bundles (Fig 2 row 2), which are the CT, PMs, BB with right (BBR) and left (BBL) sides and the LFO between both atria. From the information provided by the anatomical observations, the architecture of preferential bundles in the new model is the following: i) the CT runs down longitudinally from the SAN to the vestibule of the TV, with dimensions of 6 cm x 0.3 cm x 0.09 cm (length, width, intramural maximum thickness); ii) 10 PMs were included along the CT as parallel ridges that run on the endocardium of the RLW [16]. Their dimensions range from the smallest PM (1.7 cm x 0.15 cm x 0.06 cm) to the largest (4.5 cm x 0.3 cm x 0.06 cm). The septum spurium is an additional PM that branches out into 9 smaller PMs (each 0.1 cm wide) covering the endocardial surface area of the RAA; iii) the antero-superior interatrial connection follows the tubular structure of the BB. The BBR section originates in the SAN and extends 3.5 cm towards the LA with a width varying between 0.2 and 0.9 cm. The BBL turns around the LAA splitting in two branches. First branch (2 cm x 0.3 cm) extends towards the lower face of the LAA, and the second one (3 cm x 0.6 cm) extends around the LAA on its upper side, following the experimental observations and other previous results [17].

Atrial fibre orientation was analysed in detail to determine the number and location of regions with a common fibre organization (Fig 2 row 3). This orientation was assigned to each region as the cross product between the principal region-defining vector direction and the normal to the external surface of each element belonging to that region. The result of this product was defined as the longitudinal direction of the fibre. As can be seen, the BB (Fig 2, row 3, column a), CT and PMs (Fig 2, row 3, column c) and the RLW (Fig 2, row 3, column d) match the histological observations (Fig 1). It is important to note that several neighbouring regions show large differences in the principal direction of their fibres and thus strongly influence the propagation of the electrical wavefront. These include: CT vs RLW, CT vs PMs, RLW vs TV, BBL vs LSW, and LPW vs base of LPV and vs RPV. All the anatomical properties and the morphology of each atrial region are described in detail in S1 Table.

### C. Cellular and tissue electrophysiology modelling

Together with fibre orientation, regional electrical differences in cellular and tissue properties are critical characteristics for obtaining a realistic atrial action potential (AP) morphology and activation/repolarisation sequence. In our study, the cellular electrical activity is modelled by the Maleckar ionic model [11]. The maximum conductance of three ion currents I_to_, I_CaL_ and I_Kr_ was adjusted in eight different regions of both atria (RA, PM, CT, BB, TV, LA, PV, LAA and MV), see Table 1, considering the experimental observations on AP morphology and duration [18–22]. A similar methodology was also used in previous computational works [7,8,23].

**Table 1:** Ionic channel conductance and APD90.

Ionic Conductance / model			RA, PM	CT, BB	RAA	TV	LA	PV	LAA	MV
**g**_**to**_			1.00	0.50	1.35	1.35	1.00	1.35	1.35	1.35
**g**_**CaL**_			1.00	1.00	1.00	0.80	0.67	0.67	0.67	0.53
**g**_**Kr**_			1.00	0.50	1.00	2.00	1.60	3.20	1.60	3.20
**Simulated APD**_**90**_ **(ms) BCL = 1Hz**			199.0	227.0	189.0	173.0	181.0	161.0	171.0	158.0
**Experimental APD (ms)**	**APD**_**95**_	[18]	190.0±3	270.0±10	180.0±3	160.0±4				
	**APD**_**90**_	[19]	170.9±4.3				152.9±4.3			
	**APD**_**90**_	[20]					200.0	178.0		
	**APD**_**90**_	[24]	207.0±18							

Relative values for the ionic channel conductance in each model and the APD90 after stimulating the cell model during 60 minutes. Experimental values from canine and human cells are also included.

**Fig 3:**
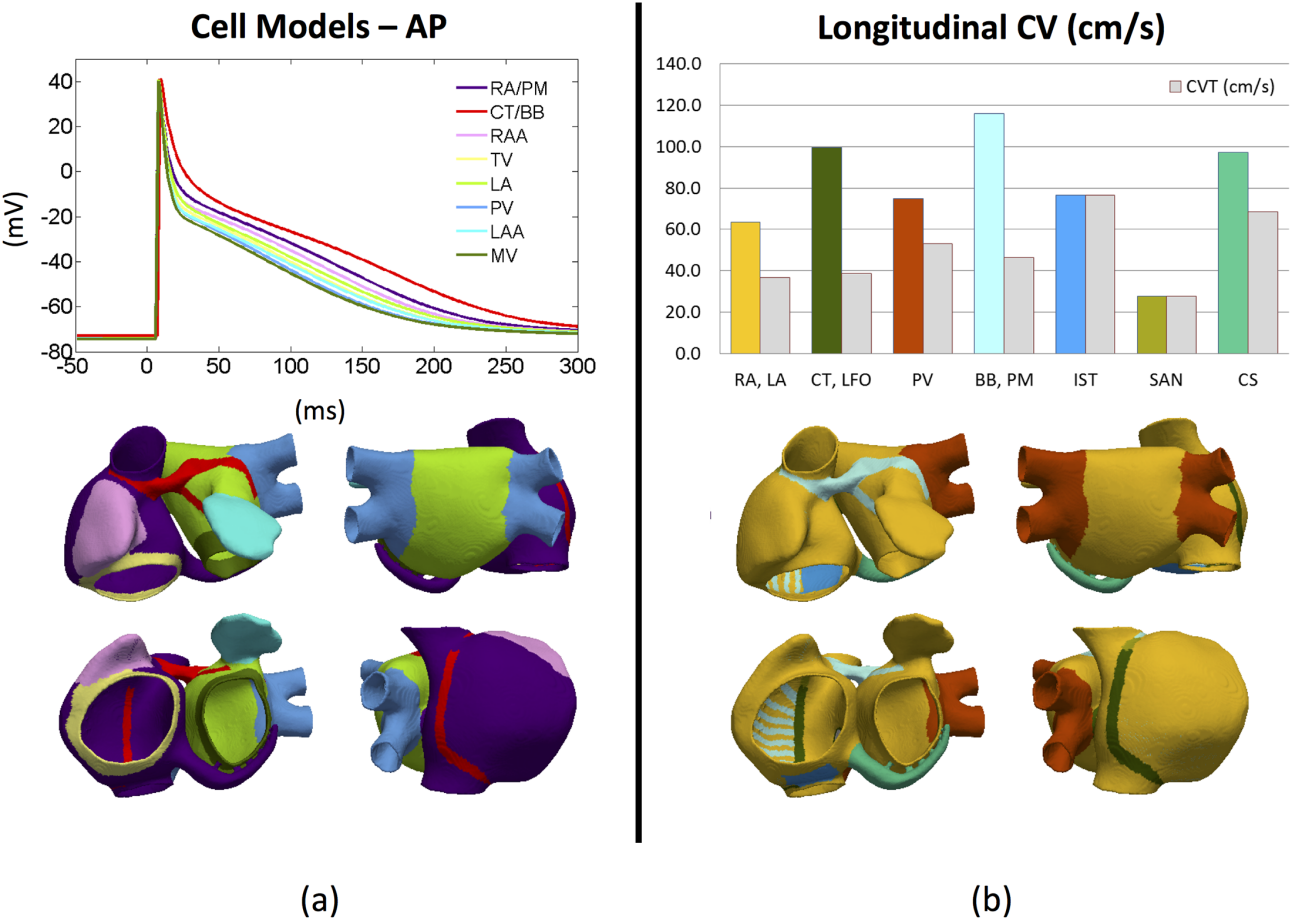
Cellular and tissue properties of the atrial model. a) Action potentials produced by each cell model in 0D (top) and the atrial regions to which each model has been assigned labelled by colour (bottom); b) Longitudinal (coloured) and transversal (grey) tissue conduction velocities in 3D (top) and the corresponding atrial regions colour coded (bottom). Each region has different longitudinal tissue conductivity.

To stabilize the cellular model of each region, a train of 3600 stimuli (1 ms of duration and 52 pA/pF of amplitude) was applied at a basic cycle length (BCL) of 1000 ms. After stabilizing, an additional stimulus with the same BCL was applied to check whether intracellular concentrations of [Na^+^], [K^+^] and [Ca^2+^] remained stable, obtaining a mean variation lower than the 2% millimolar measured instantly before the depolarization between the first and last stimulus within the last 60 stimuli. Fig 3a shows the characteristic AP for each of the 8 regional cell models. The differences in APD_90_ produced by each model can be found in Table 1 together with experimental values. For the RA and the PM, the APD_90_ (199 ms) is similar to values measured in human subjects (207.0±18 ms) at the same BCL of 1 Hz [24]. The models for the CT, the BB, the RAA and the TV regions were adjusted to match the experimentally observed APD_90_ ratios between different atrial regions within the RA The ratios obtained in the models were: +14.4% for CT/BB, -5.0% for RAA and -13.1% for TV, which have been experimentally reported as +42.1%, -5.3% -15.8%, respectively [18]. Our LA model has an APD_90_ ratio of -9.0% with respect to RA/PM versus an experimental observation of -10.5% [19], and a -11.0% reduction of the PV versus the LA as observed by [20]. Due to the lack of physiological data related to the LA, a similar variation between the RA and the LA was applied to the LAA with respect to the RAA (-9.5%) and the MVR with respect to the TVR (-8.7%). These eight cellular models were then assigned to specific regions within the atria (see S1 Table for full details).

The conduction velocities in each region were tuned by adjusting the longitudinal conductivities (σ_L_) and the anisotropy ratios (σ_T_/σ_L_) in a slab model (50x50x3 linear hexahedra with the same spatial resolution as the atrial model, 300 μm). As shown in Fig 3b, the mean longitudinal conduction velocity varies between high values for BB, PM, CT, LFO and CS (from 99.5 to 116.0 cm/s), medium values for PV and IST (from 75.0 to 76.6 cm/s) and medium-low values for RA and LA general tissue (63.3 cm/s). Note that the conductivity values obtained from the slab simulations were subsequently fine-tuned in the atrial model to match atrial activation sequences and P-wave morphologies to experimental measurements [3,25]. The anisotropy ratio (σ_T_/σ_L_) assigned to the CT, the LFO, the BB and PM (fast conducting structures) was considered very high (0.15), matching experimental observations with anisotropy ratios between 0.1 [26] and 0.21 [27]. An anisotropy ratio of 0.35 was assigned to RA and LA tissue and of 0.5 to PV and CS regions [7,8]. Finally, a ratio of 1.0 (isotropy) was assigned to IST, SAN and FO. These tissue properties determine the conduction velocity in each part of the atria, with values ranging between 63.3 cm/s and 116.0 cm/s, which matched well with the mean conduction velocity of 88±9 cm/s [16], the ranges of 70–90 cm/s through the RA free wall or 120–140 cm/s through the CT [28] and the mean RA and LA values of 70.2±9.9 and 77.0±10.8 cm/s respectively [29] measured experimentally. The complete list of atrial regions, conduction velocities and anisotropy ratios are summarised in S1 Table.

### D. 3D Torso Model

**Fig 4:**
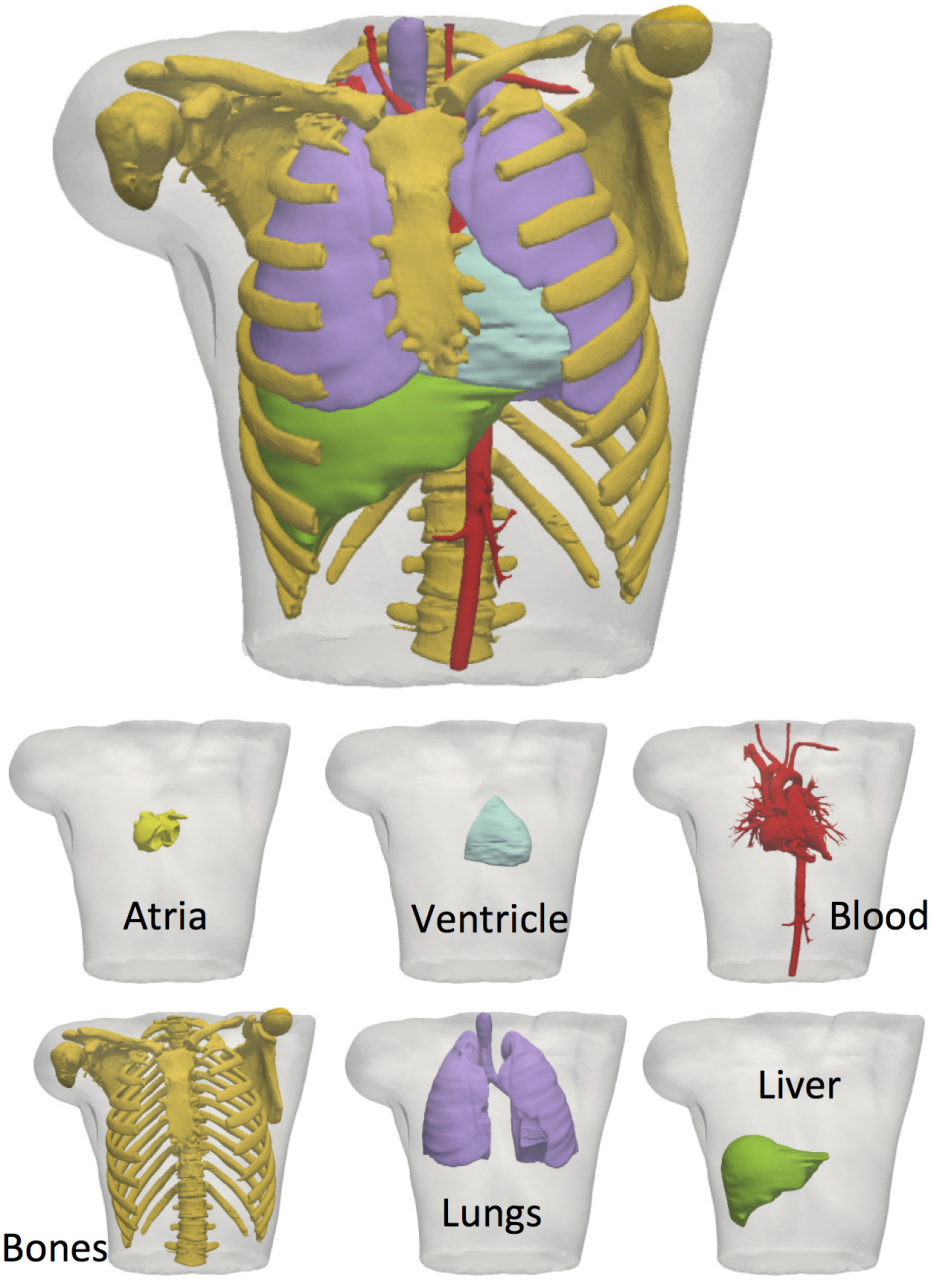
Torso model including the most relevant organs segmented from an MR stack. Included organs are: ventricle (light blue,), ii) bones (orange), iii) liver (green), iv) lungs (purple), v) blood (red) and v) chest (transparent white).

The torso dataset was obtained from the online open repository at the Centre for Integrative Biomedical Computing (CBIC) from University of Utah [30]. Data in this repository was anonymised and open to the research community at the time of building the new 3D torso model (RIUNET, http://hdl.handle.net/10251/55150). The principal organs contained in the torso (lungs, bones, liver, ventricle, blood pools, and flesh) were reconstructed by grey-level threshold segmentation to obtain the corresponding surfaces, which were manually corrected and smoothed (Fig 4). The torso was segmented by Seg3D software [31] and meshed with TetGen [32]. The resulting mesh had 190.804 nodes and 1.149.531 tetrahedral elements with a spatial resolution of 0.6 mm. Each element of the model was then automatically labelled as lung, bone, liver, ventricle, blood, and general torso, to assign their corresponding organ conductivities as reported in previous studies [30,33–37]. The model was considered isotropic and had the following conductivities: i) myocardium (4.589 mS/cm), ii) bone (0.200 mS/cm), iii) liver (0.277 mS/cm), iv) lung (0.389 mS/cm), v) chest (2.390 mS/cm) and vi) blood (7.0 mS/cm).

In this process, the original atrial model of the torso was removed and replaced by our detailed anatomical atrial model by rigid registration. The tetrahedral elements of the torso that overlapped with the atrial myocardium were highly refined to increase the number of nodes at which the extracellular potential was calculated. In this refinement process, a set of 33.905 additional torso nodes was included at a constant distance from the atrial boundary (0.8 mm) to reduce mathematical errors in the approximation of the extracellular potentials.

### E. Propagation and body potential maps

The electrical propagation of APs in the atria was described by the monodomain model:

$$\nabla \cdot \left(D\nabla V\right)=C_{m}\cdot \frac{\partial V_{m}}{\partial t}+I_{ion}\;\;in\;{\text{Ω}}_{H}$$ (1)

$$n\cdot \;\left(D\nabla V\right)=0\;\;in\;\partial {\text{Ω}}_{H}$$ (2)

where *D* is the equivalent conductivity tensor, *V*_*m*_ is the transmembrane potential, *I*_*ion*_ is the transmembrane ionic current that depends on the cellular model, *C*_*m*_ is the membrane capacitance and Ω_H_ is the heart domain.

After performing atrial simulations and obtaining the transmembrane potential, *V*_*m*_, the extracellular potentials were calculated at the torso nodes located at exactly 0.8 mm from the atrial boundary using the equations:

$$\begin{array}{l} V\left(\vec{r}\right)=-\frac{\gamma }{4\pi }\cdot \frac{\sigma_{i}}{\sigma_{e}}∭{\vec{\nabla }}^{\prime }V_{m}\left({\vec{r}}^{\prime }\right)\cdot {\vec{\nabla }}^{\prime }\left[\frac{1}{\left|{\vec{r}}^{\prime }-\vec{r}\right|}\right]dv \end{array}$$ (3)

$$V_{0}\left(\vec{r}\right)=V\left(\vec{r}\right)$$ (4)

where, $V\left(\vec{r}\right)$ is the extracellular potential calculated at point $\vec{r}$, σ_*i*_ and σ_*e*_ are the intracelular and extracelular conductivities, γ is a scale factor and $V_{m}\left({\vec{r}}^{'}\right)$ is the transmembrane potential.

Under quasi-stationary conditions [38], the torso volume can be considered as isolated. Hence, the propagation of the extracellular potential throughout the torso is governed by the Laplace equation subjected to homogenous Neumann boundary conditions

$$\nabla \;\cdot \;\left(D_{T}\nabla V_{T}\right)=0\;\;in\;{\text{Ω}}_{T}$$ (5)

$$n_{T}\;\cdot \;\left(D_{T}\nabla V_{T}\right)=0\;\;in\;\partial {\text{Ω}}_{T}$$ (6)

where *D*_*T*_ is the heterogeneous conductivity tensor of the torso, *V*_*T*_ is the extracellular potential propagated through the torso, Ω_T_ represents the torso domain and δΩ_T_ its external surface.

The monodomain Eqs (1) and (2) were solved using the operator splitting numerical scheme with ELVIRA software [39] with a constant time step of dt = 0.02 ms. The Laplace equation (Eqs 5 and 6) governing the propagation of the extracellular potentials throughout the torso was solved using ABAQUS (Dassault Systèmes Simulia Corp.), so that we obtain the potential for all the nodes in the torso domain every 1 ms. The volumetric conductor thus allowed us to study the potential maps within the torso, and at the surface, where P-waves and surface potential maps are registered (Body Surface Potential Maps, BSPM).

In the computations in which the torso was considered as uniform and infinite, Eqs (3) and (4) were used to calculate the pseudo-ECG in the precordial and standard leads from the simulated transmembrane potential, *V*_*m*_.

## Results

### A. Multi-scale atrial activation

**Fig 5:**
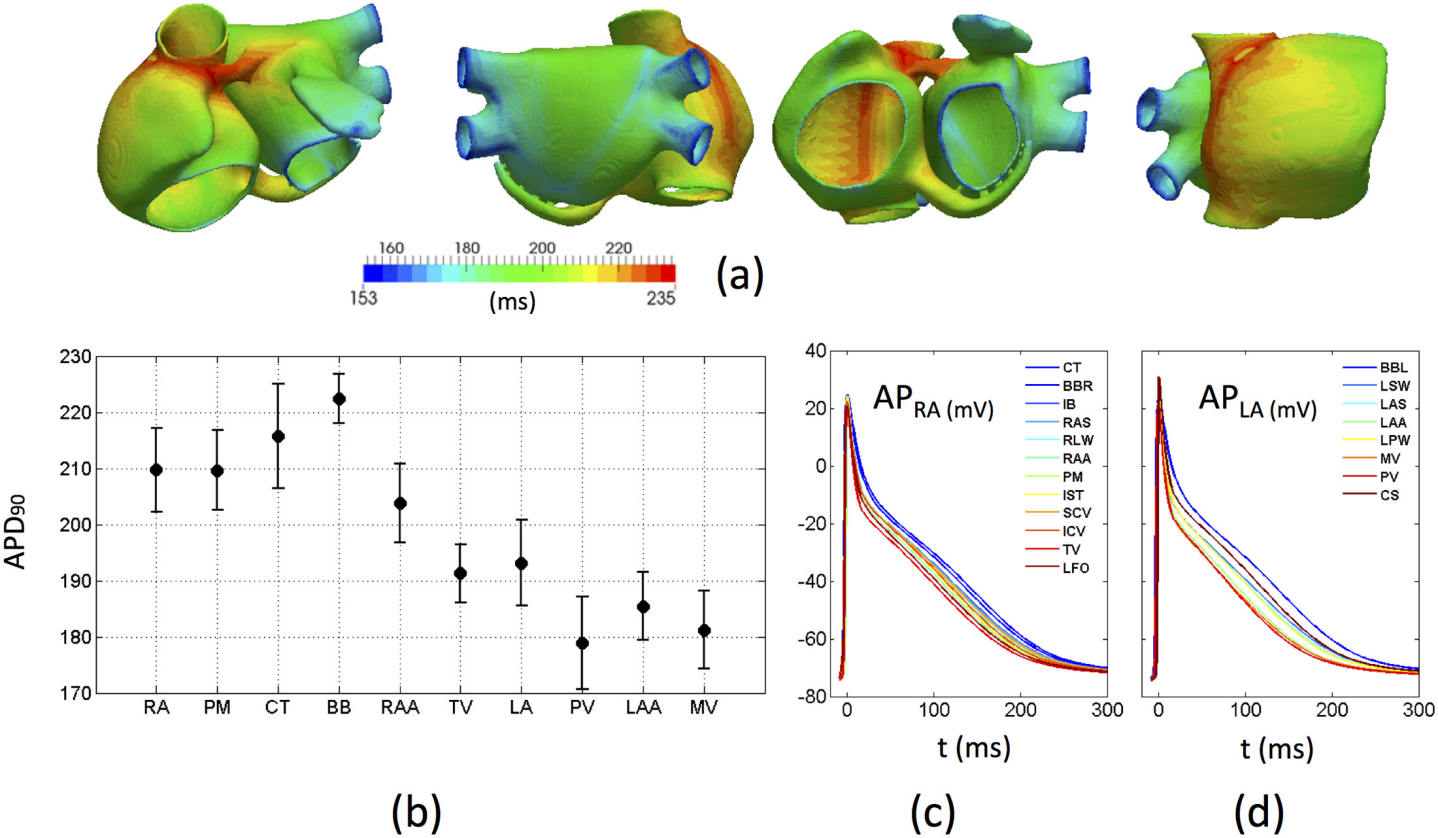
Atrial action potential (AP) morphology and duration. a) APD_90_ (ms) measured in each node of the atria at the tenth beat; b) mean and standard deviation of the APD_90_ (ms) for each region; AP (mV) registered at one random node from c) each structure of RA, and d) each structure of LA.

The eight stabilized cellular models (see Methods) were coupled into the 3D atrial model. Next, 10 additional stimuli with a frequency of 1 Hz, amplitude of 30 pA/pF and duration of 2 ms were applied to the SAN region to reach the steady-state in 3D and smooth differences between neighbouring regions. Fig 5a shows the spatial variability of APD_90_, which ranges from the lowest values at the PVs (153 ms) to the highest at the BB (235 ms). Fig 5b shows the mean APD_90_ and first standard deviation (σ) for each atrial region where minimum mean values correspond to the PVs (178.9±8.2 ms), whereas maximum mean values are from the BB (222.4±4.3 ms). Dispersion within each region ranges from σ = ±9.3 ms (CT) to σ = ±4.3 ms (BB). The relationship between APDs in each region at organ scale (Fig 5c and 5d) was maintained when compared to 0D, although a smoothing effect was observed due to coupling as expected (Fig 3a).

**Fig 6:**
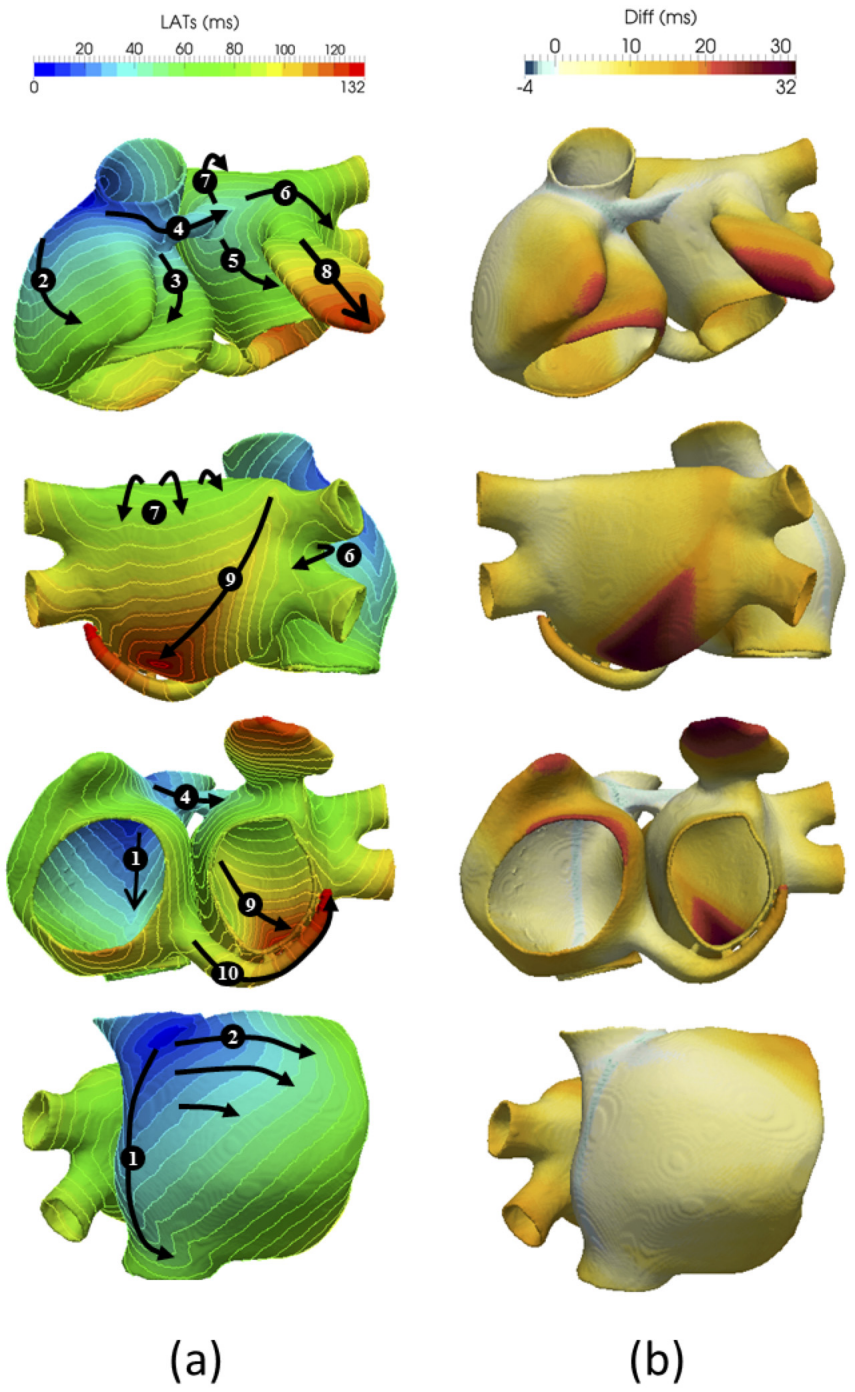
Atrial local activation times (LATs). a) Isochronal LAT maps produced by the atria with fibre orientation (anisotropic); b) LAT differences between atrial model with anisotropic and isotropic tissue conductivity.

Fig 6a shows the isochronal map of the atria activation sequence for the last stimulus applied (the tenth). After the SAN is depolarized (t = 0 ms, dark blue regions in Fig 6a), the CT, the BBR, the IB and the SCV simultaneously start their respective depolarisations (light blue regions in Fig 6a). The activation descends rapidly through the CT towards the ICV for 57 ms (arrow 1) activating sequentially the PMs and the endocardial wall of the IB. This activation pattern produces a triangular wavefront that spreads from CT to TV through the RLW and the RAA for 85 ms (arrow 2). The right side of the atrial septum (RAS) is activated by the IB at t = 20 ms, (light blue regions in Fig 6a), which allows a second wavefront to spread with lower conduction velocity from CT towards TV (arrow 3). Both wavefronts (arrows 2 and 3) collide in the TV, the last RA region activated, and thus produce the last activation time registered in the RA at t = 109 ms (orange regions in Fig 6a).

The activation moves through the BBR from the RA to the LA (arrow 4), registering the first activated point in the LA at t = 38 ms (light blue regions in Fig 6a). As can be seen, the original LA wavefront splits up into two parts, which facilitates the rapid depolarization of the LSW and regions around the LAA. The left side of the atrial septum (LAS) is partially activated by the BBL (arrow 5) and partially by the LFO 10 ms later (not visible in the figure). These LA activations produce four wavefronts at t = 64 ms, as can be observed in Fig 6a, which advance simultaneously towards the MV (arrow 5), the union between the superior and inferior RPV (arrow 6), the LPW (arrow 7) and the LAA (arrow 8).

The LAA shows a slow conduction velocity, since the depolarization wavefront travels perpendicularly to the local fibre direction, taking 61 ms to be completely depolarized. In the posterior wall, the LPW depolarization sequence depends on two wavefronts, one arriving from the LSW (arrow 7) and the other crossing the RPV (arrow 6). This causes a diagonal wavefront (arrow 9) that electrically sweeps the posterior wall from the superior RPV towards the inferior LPV for 64 ms.

The CS (arrow 10) is depolarized together with the LPW but with a slower conduction velocity due to the circular fibre direction. The latest activation time registered in the atria corresponds to t = 132 ms and is concurrent with the latest activation of the CS.

A complete atrial activation sequence together with all the time points that define the first and last activation of each atrial region can be seen in S1 Video and S1 Table.

In order to quantify the contribution of fibre orientation to the activation sequence, we compared the previous results with the atria activation sequence obtained from an isotropic atrial model. Tissue heterogeneity and longitudinal conduction properties were kept identical for both models, but the anisotropy ratio was fixed to 1:1 for the whole atria. Fig 6b shows the absolute differences in local activation times (LAT) between both configurations. Positive differences (reddish colours) correspond to regions that activate earlier in the isotropic model, whereas negative (bluish colours) correspond to regions that activate earlier in the anisotropic model. Since longitudinal conductivity and heterogeneity are equal in both models, the activation sequences seem similar, although the wavefront spreads out almost circularly from the SAN in the isotropic model, thereby the depolarization times registered are considerably lower in specific parts of the atria. The strongest effect produced by the isotropic model is the increase in the wavefront velocity in these regions, where it advances mainly perpendicular to the longitudinal fibre direction, such as the atrial appendages (RAA and LAA), the TV and the lower right area of the LPW. In the isotropic model the latest activation times are t = 94 ms and t = 112 ms for the RA and the LA respectively, 12.5% and 15.2% faster than in the anisotropic atrial propagation.

### B. Body Surface Potential Maps and ECG

**Fig 7:**
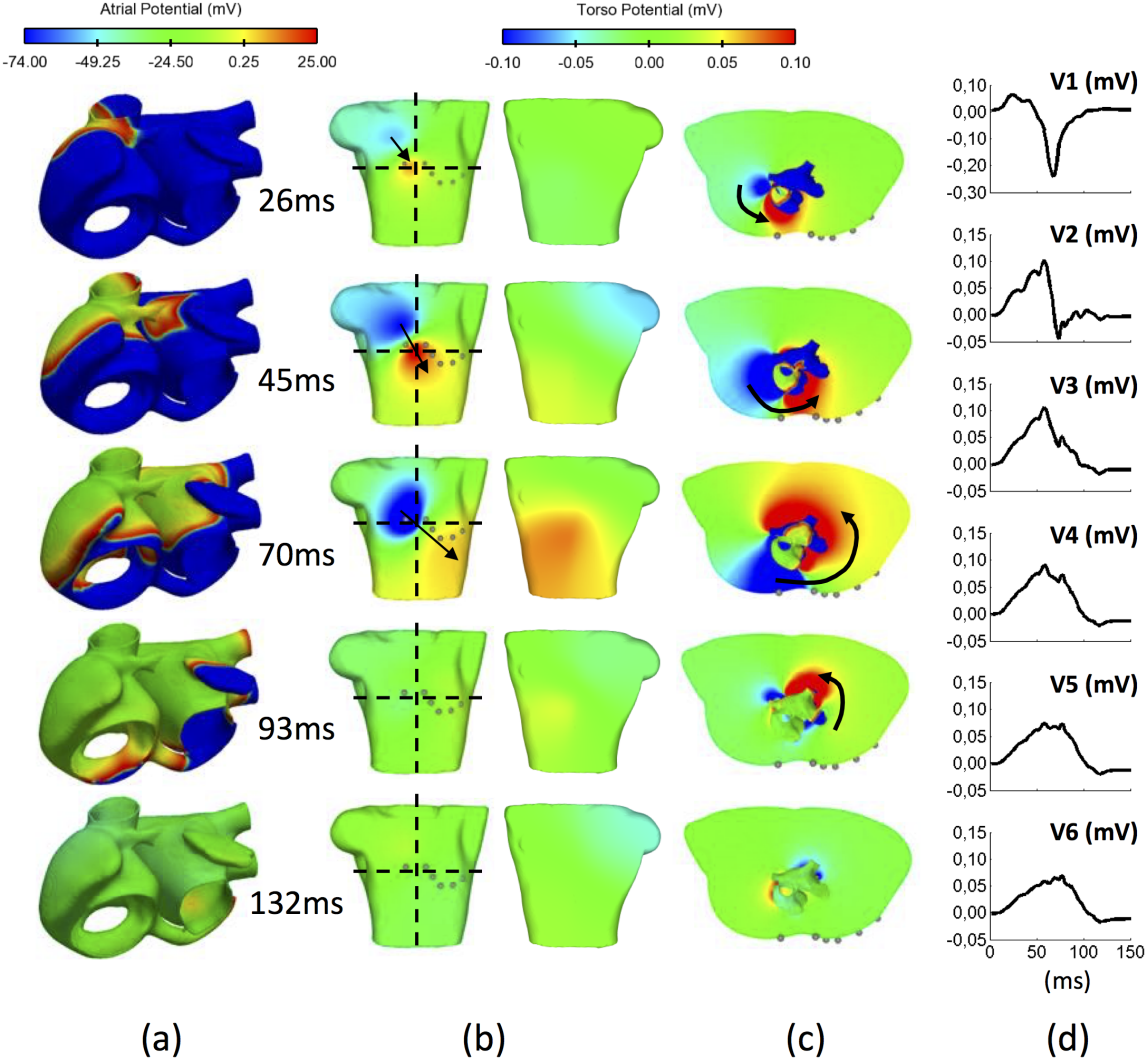
Body surface potential maps over time. a) Atrial activation sequence at characteristic time points (first contact with BB at t = 26ms; starting depolarization of LA at t = 45ms; rotation of the atrial wavefront at t = 70ms; final RA depolarization from t = 93ms; latest LA depolarization time at t = 132ms); b) Torso frontal (left) and rear (right) surface displaying the electrical dipole at the same instants; c) Axial torso plane at the V1 and V2 height; d) P-wave (mV) registered at the precordial leads V1 to V6.

The distribution of potentials in the torso at different time points of the atrial activation is shown in Fig 7 on coronal and axial planes (see S2 Video for the complete atrial and torso propagations) where the characteristic electrical dipole is clearly visible. During SAN depolarization (t = 0 ms, not shown in the figure) the atria and torso remain in a resting state. At t = 26 ms there is a visible dipole located on the upper right quadrant of the frontal chest (Fig 7a and 7b). For the following 44 ms the dipole slightly rotates towards the lower left quadrant and then backwards (at t = 70 ms) until it completely disappears from the surface when the RA is almost depolarized (at t = 93 ms). This rotation is clearly visible in the axial plane (Fig 7c). Atrial depolarization produces the maximum potential registered on the torso surface at t = 45 ms, when the atrial wavefront is travelling across the RLW and the RAS towards the upper side of the TV, and the interatrial wavefront starts the activation of the LA through the BB. At t = 70 ms, the electrical propagation flows towards the isthmus on the RA, and the left superior wavefront on the LA splits into three different directions (Fig 6a, arrows 5 to 7), causing dipole rotation and forcing higher potentials towards the posterior side of the torso. At t = 93 ms, the RA is almost depolarized but some regions in the LA such as the LAA, the MV, the CS and the LPV are still in a resting state. From then until t = 132 ms the potentials registered on the surface start decreasing, as can be observed in Fig 7b and 7c.

P-waves with a negative single-phase are obtained on the upper right chest (Fig 7d) precordial lead V1), and a positive single-phase on the inferior anterior torso (Fig 7d) precordial leads V3 to V6). Biphasic waveforms with initial positive deflection are observed from leads on the central and upper parasternal region (Fig 7d, precordial lead V2).

**Fig 8:**
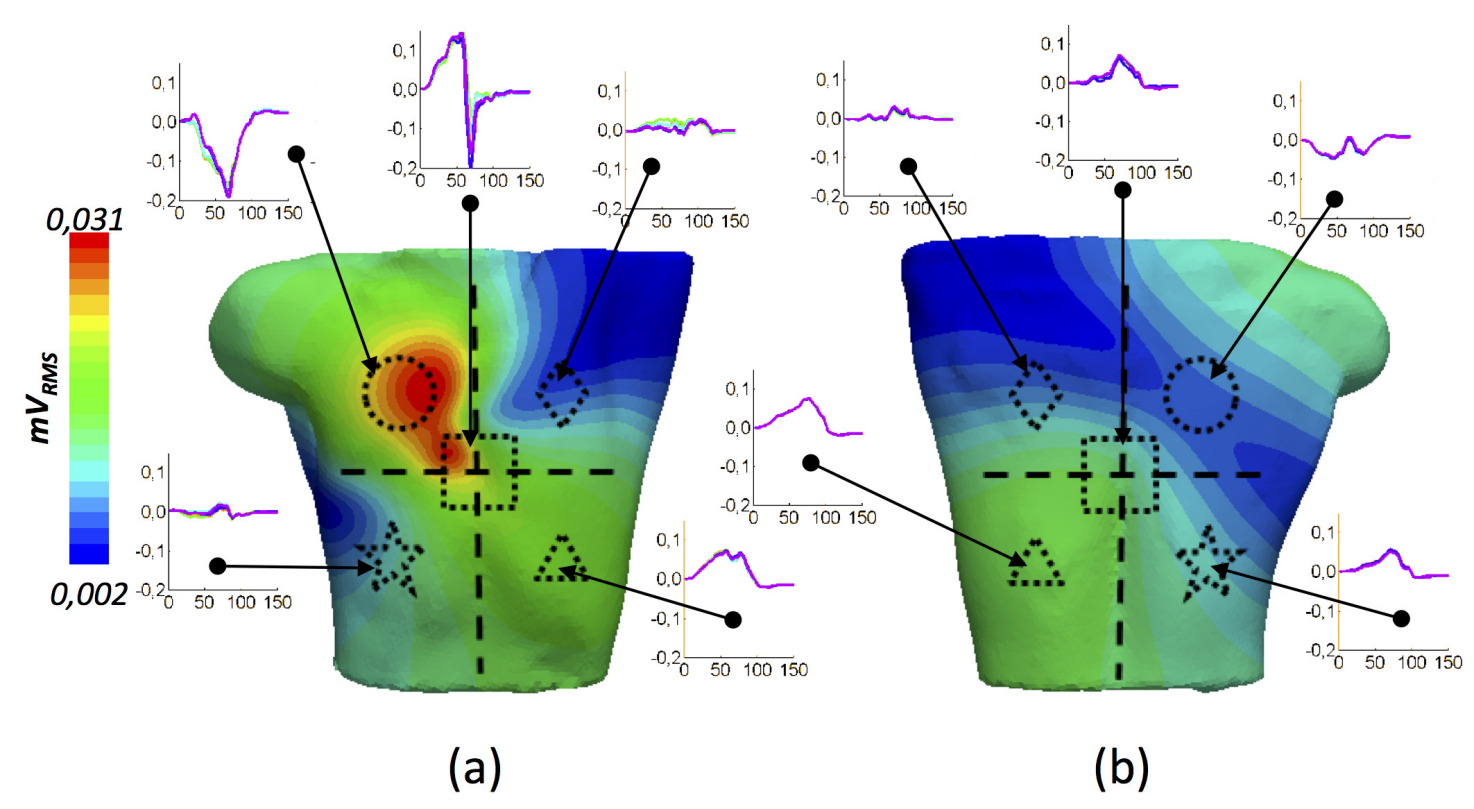
Spatial information from the potential root means square (B-RMS). a) B-RMS (mV_RMS_) at the frontal torso view; b) B-RMS (mV_RMS_) at the rear torso view. Geometric forms in each quadrant represent the torso area where the displayed P-waves are registered (units: mV *vs* ms).

We performed a deeper signal analysis computing the Root Means Square (RMS) on the whole BSPM (from now on B-RMS) to obtain additional information on the P-wave signal magnitude and its morphology (Fig 8). The signals registered within the dotted square at the central position (high B-RMS values around 0.031 mVrms) correspond to the region of maximum potentials and have very similar biphasic morphology (0.35 mVpp). The main characteristic of all the P-waves within this square lies in their biphasic morphologies with a turning point from positive to negative potentials at t = 70 ms. This morphology is due to the rotation of the electrical dipole shown in Fig 7. P-waves in the upper right quadrant (dotted circle) also have the highest values of B-RMS, but show monophasic patterns with high negative amplitude (0.22 mVpp). A negative to positive change in the signal slope is observed in all the signals registered within this circle at t = 70 ms, also produced by the rotation of the electrical dipole. P-waves from the upper left quadrant (dotted diamond) and the lower right panel (dotted star) present low values of B-RMS (0.002 mVrms) and a very noisy monophasic morphology with low amplitude (0.04 mVpp and 0.03 mVpp, respectively) that prevented us from inferring useful information related to atrial activation. Finally, in the lower left quadrant (dotted triangle), we registered P-waves with medium values of B-RMS, indicating once again signals with sufficient monophasic amplitude (0.10 mVpp) to be considered useful information. As in the previous cases, the double peak at t = 70 ms (M-shaped morphology) represents the rotation of the electrical dipole. Potential B-RMS distribution in the rear torso was also analysed (Fig 8b) and similar conclusions were reached. Only signals registered on the green area of the rear central (dotted square) and lower quadrants (dotted triangle and star) allowed noise-free signals to be analysed.

This analysis allows us to predict the areas of the torso areas in which P-waves have enough amplitude and sufficiently well-defined morphology to be considered as sources of information for clinical decisions or the solution of the inverse problem. Based on these results, we define the frontal upper right and the frontal and rear lower left quadrants as the best sites to register P-wave signals. The blue coloured areas can thus be discarded in the analysis of the whole BSPM.

**Fig 9:**
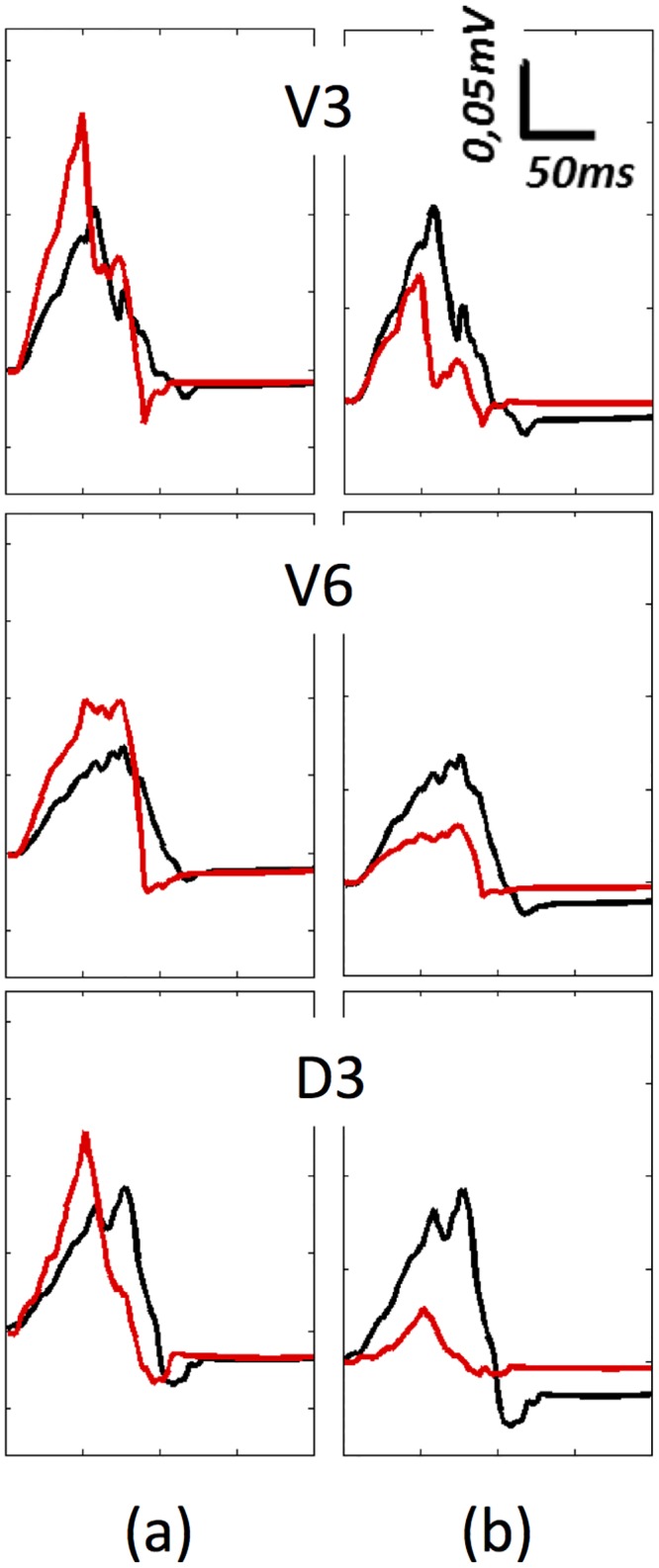
Effect of the tissue heterogeneity in the P-wave morphology. a) Differences between anisotropic (black line) and isotropic (red line) atrial tissue propagation using a complete torso model; b) Differences between a complete torso model with heterogeneous electrical conductivities and the heat transfer equation (black line) and P-waves registered considering a homogenous and infinite torso approach using the equation of extracellular potentials (red line).

In this multi-scale validation of physiological atrial activation and its propagation through the torso it is important not only to analyse the signals on the torso surface, but also to find the conditions that determine the amplitude, duration and morphology of the P-waves. Two main effects must therefore be taken into account: i) the atrial anisotropy produced by the distribution of the atrial fibres and ii) the influence of the torso as a volumetric conductor. The individual effects on the P-wave morphology produced by changes in these properties are shown in Fig 9. The left panel shows differences between anisotropic (black line) and isotropic (red line) atrial propagation (see also Fig 6). The main changes produced by an isotropic atrial model when registering at the unipolar precordial or standard leads (only shown V3, V6 and the standard lead III) are: a) reduction of P-wave duration (-38% measured on V3); b) increase of P-wave maximum amplitude (37% measured on V3); and c) simultaneous depolarization of the RA and the LA, which causes differences in the activation patterns with erratic morphologies.

In the right panel we analyse differences in P-wave morphology considering: a) a complete heterogeneous torso model, and b) the torso as a homogenous and infinite volumetric conductor (see Material and Methods, section E). The P-wave produced using the homogenous volumetric approach (red line) differs from the heterogeneous approach (black line) in three significant aspects: a) reduction of P-wave duration (-43% measured on V3); b) reduction of P-wave amplitude (-56% measured on V3) opposite to the effect produced by isotropy; and c) morphological changes.

These results reveal the importance of considering muscle fibre directions in the atrial model and tissue conductivities in the torso model. Both properties significantly influence the amplitude, duration and morphology of P-waves.

### C. Contribution of individual atrial regions to the ECG

Figs 7 and 8 show that maximum potential values and B-RMS produced by the whole atria are registered in the centre of the torso frontal surface. However, one of the biggest challenges for cardiac electrophysiologists is to assess individual contributions from each region of the atria to the complete ECG morphology. Although it is not in practice possible to divide and individualize the atrial activation sequence produced by each of the anatomical atrial structures, the use of electrophysiological modelling may help us to understand how different tissues contribute to the formation of the BSPM and the P-wave of the ECG.

**Fig 10:**
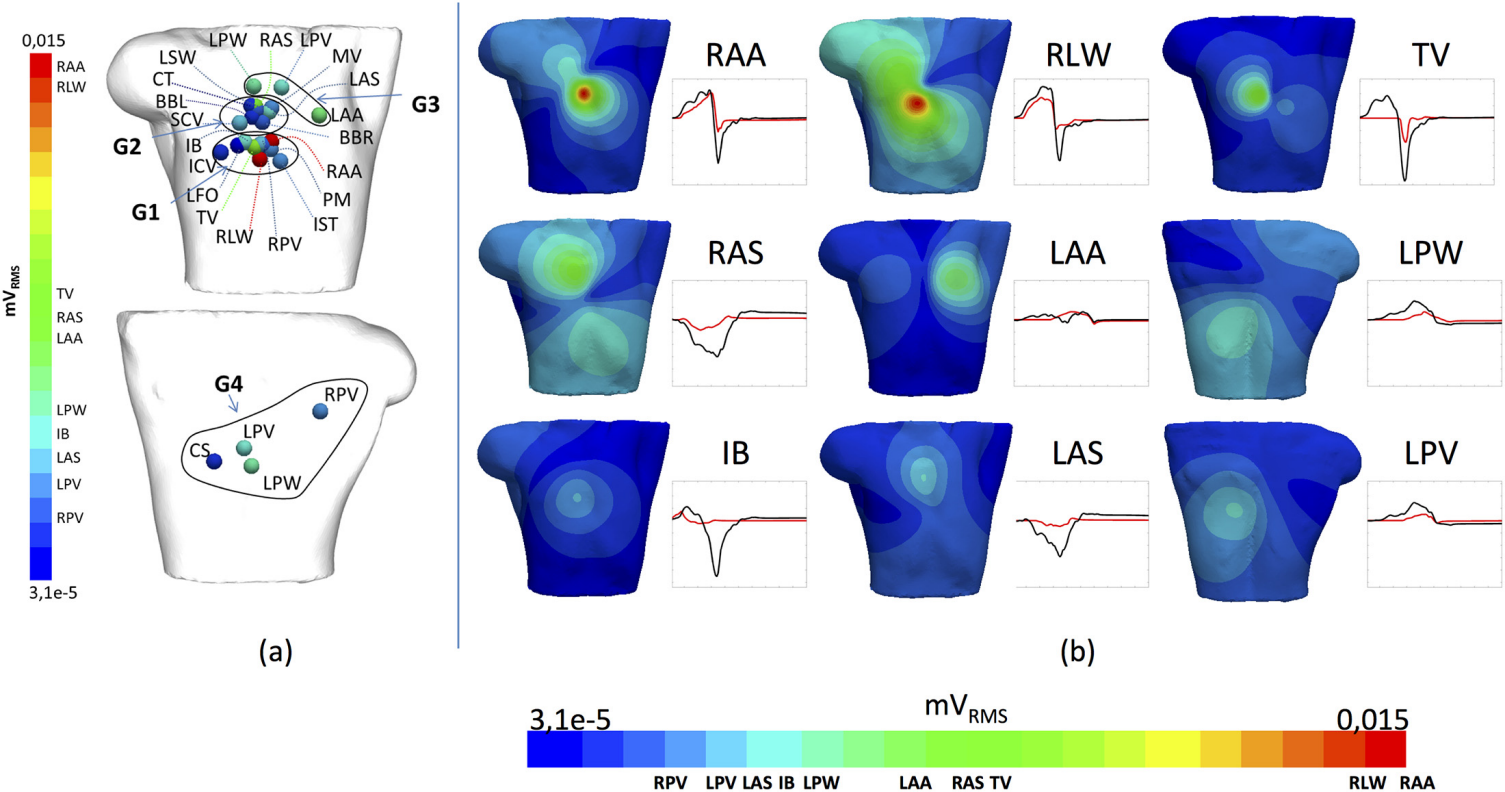
Contribution to the ECG from individual atrial regions. a) Location of the maximum value of potential RMS produced from each atrial structure; b) B-RMS patterns (mV_RMS_) from the individual atrial structures with the highest contributions. Also displayed the P-waves (mV *vs* ms) registered at the point with maximum potential RMS value (red line) compared with the total P-wave registered at the same point (black line).

**Fig 11:**
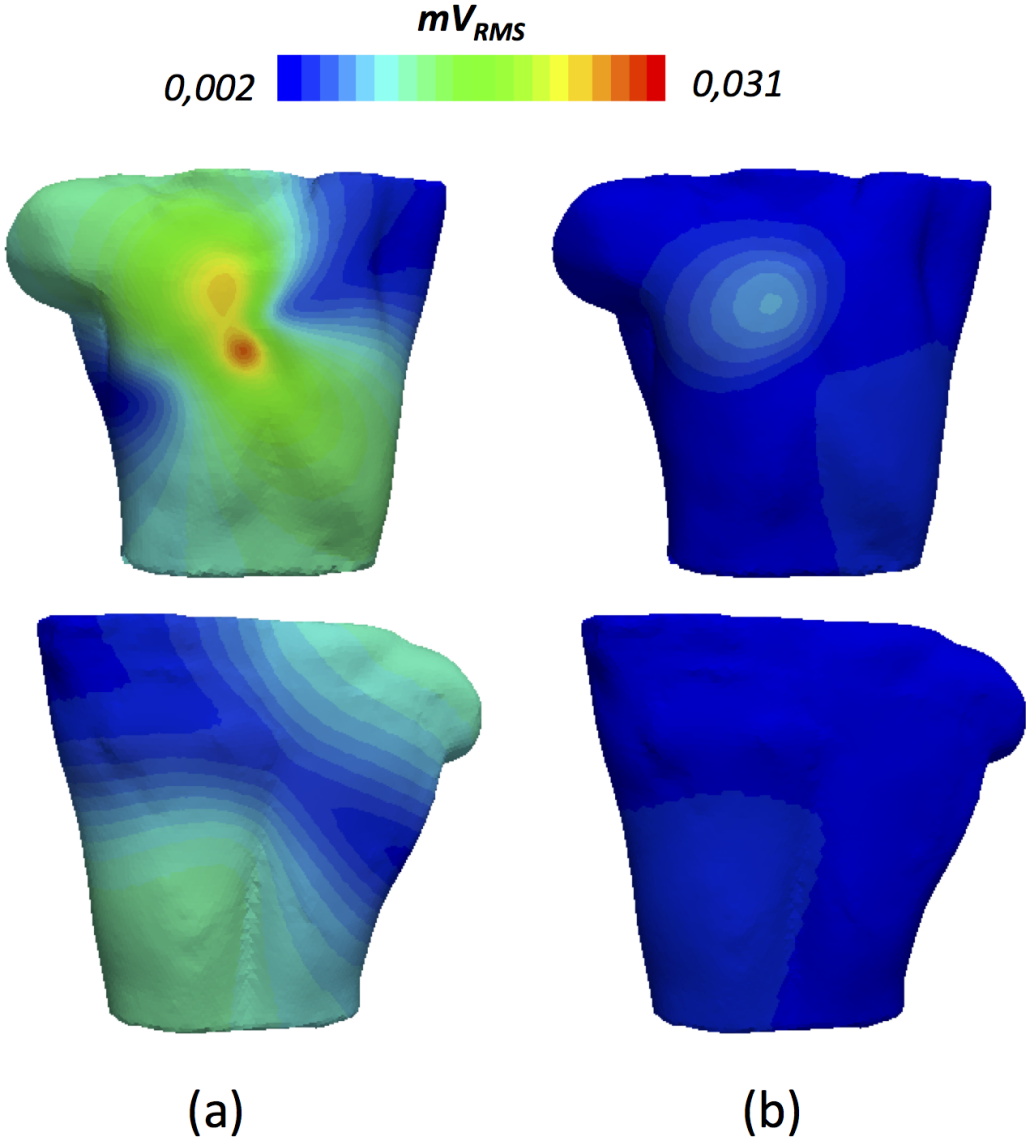
Contribution of the most influential individual atrial regions to the total RMS distribution. a) Joint contribution of the signals of the 9 regions jointly responsible for the 89% of the total contribution (RAA, RLW, TV, RAS, LAA, LPW, IB, LAS and LPV); b) Joint contribution from the remaining twelve regions responsible for the 11% of the total contribution.

As it was possible to isolate the contribution of each atrial region by masking the electrical contribution of other regions, we analysed the individual contribution to the B-RMS potential and corresponding P-wave produced by each atrial region in sinus rhythm. Fig 10a shows the torso surface area in which the different atrial regions present their maximum contribution to B-RMS maxima. Four spatial clusters can be identified: the G1 group (the lowest position of the frontal torso) with contributions from: RAA, RLW, TV, IB, PM, IST, RPV, LFO and ICV; the G2 group (the middle position) with contributions from: RAS, LAS, LSW, CT, BBL, SCV, BBR, MV; the G3 group (the upper position) with contributions from LAA, LPW and LPV; and the G4 group with contribution from regions with better registry on the back: LPW, LPV, RPV and CS. G1 shows the highest values produced by RAA and RLW, as confirmed from the amplitude of the individual P-wave (in red) related to the total P-wave (produced by the whole atrium) registered at the same point (in black). Should we consider the whole range of potential RMS and the B-RMS maps from each region, only 9 atrial structures/regions (RAA, RLW, TV, RAS, LAA, IB and LAS on the frontal view and LPW and LPV on the rear view), produce a significant potential contribution with very high (red) to medium (green) RMS values, as can be seen in Fig 10b. The other 12 atrial structures produce individual homogeneous body maps with RMS (blueish) values lower than 20% of the maximum contribution when considering the whole atria (S1 Fig shows the B-RMS produced by each of the 21 atrial regions). Fig 11a shows the potential RMS patterns obtained by adding the contribution of these 9 regions jointly, which are responsible for the 89% of the total contribution produced by the 21 regions (Fig 8). The other 12 structures, Fig 11b, are responsible for the remaining 11%, suggesting that the morphology of the total P-wave in sinus rhythm depends mainly on the 9 identified regions (even when they only represent the 65% of the total atrial volume).

**Fig 12:**
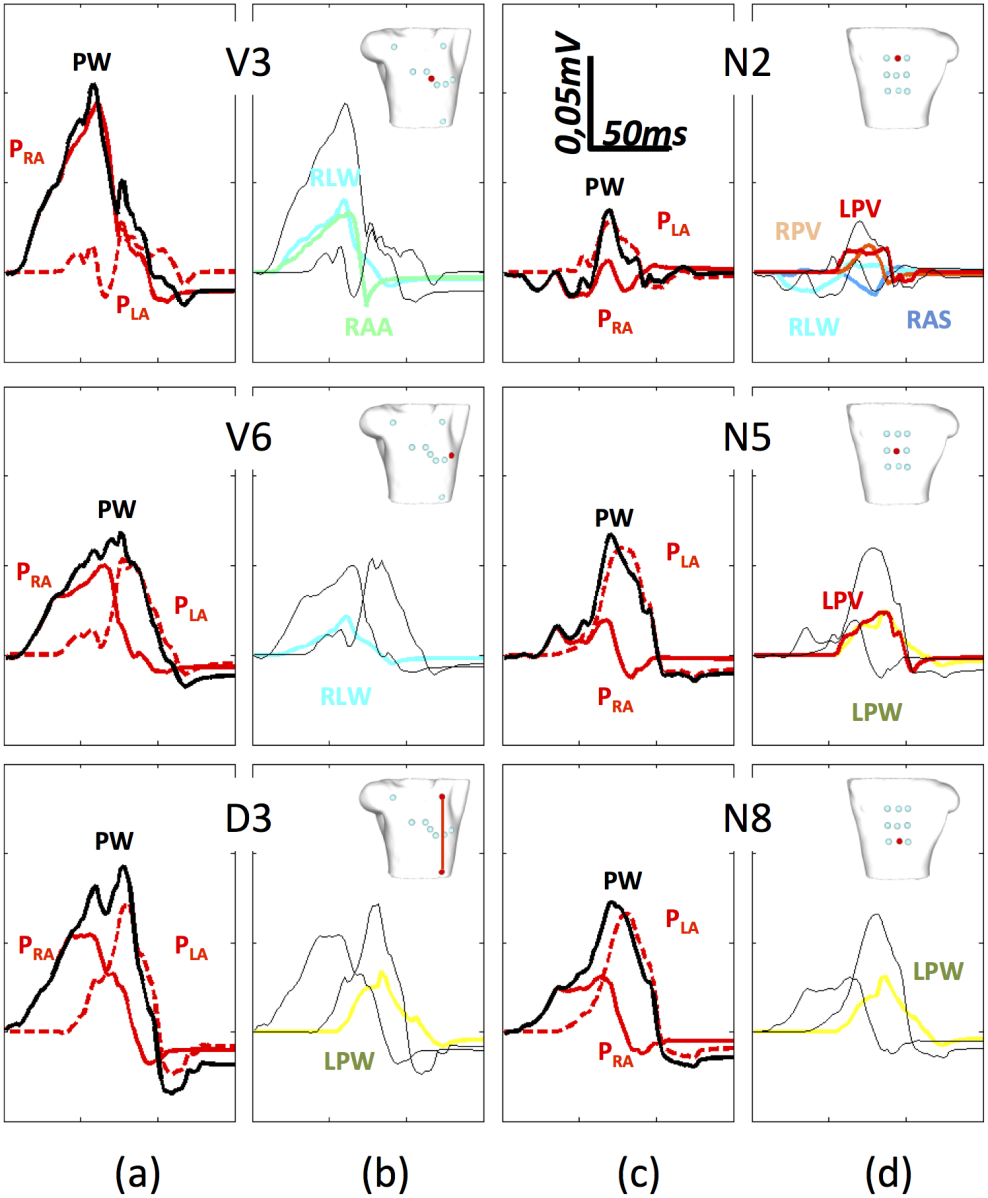
P-waves registered on the torso precordial (V3 and V6) and standard (D3) leads. a) and c) represent the P-wave produced by the whole atria depolarization (black line), the contribution from the RA structures (continuous red line) and the contribution from the structures in LA (dotted red line). b) and d) show only those individual regions with maximum amplitude of at least the 30% of the maximum P-wave amplitude registered at the frontal and rear sides.

These individual contributions were also analysed with respect to different positions of the electrocardiographic leads. Fig 12 shows the signals registered in precordial leads V3 and V6, the standard lead III, and on the rear side N2, N5 and N8 (see red points in detail for an exact location on the torso surface and S2–S5 Figs for the remaining leads).

The P-waves (black lines) produced by the complete atria depolarization at different standard locations are displayed in Fig 12a (torso front) and Fig 12c (torso back). The contribution from the RA structures (solid red line) and the contribution from the LA structures (dotted red line) is also depicted in the figure. Fig 12b (torso front) and Fig 12d (back) show only those individual regions whose contributions mainly determine the RA and the LA total waveforms, with a maximum amplitude larger than 30% of the maximum P-wave amplitude.

Similarly to V1 and V2 (S2 and S3 Figs), in the case of V3 the RA contribution is significantly higher than LA and determines the P-wave morphology. Within RA, the V3 waveform is mainly determined by the RLW and the RAA (blue lines) and the same occurs with lead V2. In the case of V1, the TV and the RLW contributions determine the waveform. For the LA, no individual region contributes more than 30%, hence the LA waveform is the result of a number of small contributions. The behaviour of the other three precordial leads is similar to the behaviour observed for V6 in Fig 11 (see S2 and S3 Figs for V4 and V5). In V6, the RA morphology is mainly determined by the RLW (blue line), but no particular region defines the LA. However, the P-wave is the result of comparable contributions from both atria, where RA dominates the first part of the wave (until t = 70 ms) while LA starts dominating from then to the end. Finally, when registering Einthoven leads, the resulting P-wave depends on the specific lead, although both atria overlap their effects to produce M-shaped complexes with the inflexion time at t = 70 ms. Thus, in the case of the standard lead III (D3), no individual region of the RA determines the RA waveform and only LPW (orange line) appears to significantly contribute to LA morphology. However, standard lead I (D1) is defined by the contributions of RLW plus LAA, and Einthoven lead II (D2) from those produced by RLW, RAS and LPW (see S2 and S3 Figs).

We also show in Fig 10 that LPW, RPV, LPV and CS produce their maximum potential on the back, where the contribution from LA structures is much higher. This is also consistent with the results in Fig 12d, in which, as we move towards the area of the biggest contribution, P-wave amplitude increases and the atrial structures responsible for the P-wave signal are mainly from the LA. Thus, the LPV and the LPW are the only regions defining the morphology at the central and lower rear leads N5 and N8. S2–S5 Figs show the electrical signals registered on eighteen leads on the frontal and rear view of the torso surface.

Overall, although the distance between a recording lead on the torso and a given atrial region is an important determinant of potential RMS recorded, as expected, other factors such as wavefront direction has a main effect on the polarity of the signal.

## Discussion

This paper presents a new detailed multi-scale computer model for the study of atrial electrical activation. Eight different models were adjusted on the cellular scale, allowing us to generate APs personalised to different types of atrial tissues. On the organ scale, an anatomo-functional atrial model was developed including the specialized anatomical structures of the atria, cellular and tissue electrophysiological heterogeneity, with detailed descriptions of fibre orientation. As previous studies [7,8,40–43], we decided not to consider the variability of the atrial wall thickness and its contribution to the P-wave morphology. Thus, we can analyse the isolated effect of fibre direction, electrophysiological heterogeneity and tissue conductivity on the signals registered on the body surface. On the body scale, a torso model was also built containing main electrophysiological details of the principal organs to analyse the atria’s electrical contribution to the P-wave of the ECG and to the BSPM.

This multi-scale model allowed us to study different scenarios and determine the complex sequences of atrial activation in order to investigate the regional correlation between the atrial de/re-polarization and the wavefronts propagating through the volume to the torso surface.

### A. Multi-scale anatomical model

Although a number of human atrial models already exist to study electrophysiology under physiological and pathological conditions, only a few of them present realistic geometry [8,41,44,45]. Of these, only a reduced subset consider a realistic geometry together with a detailed description of fibre orientation, specialized atrial structures and electrophysiological heterogeneities and are able to reproduce atrial activation patterns similar to those observed experimentally [8,41].

The multi-scale atria-torso model presented here improves on a number of the properties of our original computational atrial model, including: first, with regard to atrial mesh quality, hexahedral voxels were used with the higher homogeneous spatial resolution of 0.3 mm, as in [41,44,45]. Thanks to the high quality and regularity of the mesh, the stability of numerical computations is guaranteed [46]. Second, the number and size of PMs of the specialized anatomical atrial structures were redefined in line with histological observations, covering the endocardial surface of RLW and RAA, from the CT to the vestibule of the TV [15]. Additionally, the BB was lengthened to join the SAN in the RA and to hug the LAA on its left side [47]. Third, with respect to tissue anisotropy, the whole atrium was divided into 21 anatomical structures and 53 sub-regions in order to improve the local control of fibre orientation, mainly at the conduction structures (CT, PM and both sides of BB). The orientation of several regions was updated with respect to our previous model, to agree with descriptions extracted from new histological data [8]. Fourth, with respect to the cellular AP, the basic model was changed from Nygren et al. [48] to Maleckar et al. [11], since Maleckar’s model shows long-term stability in terms of restitution properties and APD_90_. The stability of these dynamic properties was considered essential to develop and validate the eight regional cell models in physiological conditions (RA/PM, CT/BB, RAA, TVR, LA, PV, LAA and MVR). All these cellular models reach steady state after approximately 10 min, although the stabilization process was carried out for 60 min, allowing robust usage at both the cell and tissue level for investigation of the human atria [49]. Fifth, with respect to tissue conductivity, different values of longitudinal conductivity and anisotropy ratios were defined as a function of the atrial region (RA/LA, CT/LFO, PV, BB/PM, IST, SAN, CS and the isolating layers of FO and inside BB). A limited number of experimental studies provide values of conduction velocities from healthy subjects in sinus rhythm, with measurements in a few human atrial structures [16,28,29]. The conduction properties presented here (conduction velocities ranging from 63.3 cm/sec in RA and LA to 116.0 cm/sec in BB and PM) were then compared and validated against experimental atrial conduction velocities, ranging from the minimum mean reported velocity of 70.2±9.9 cm/s from RA [29] to 140 cm/s maximum velocity measured at CT [28]. Experimental local activation time maps were also used to validate the atrial activation sequence. The model produced the first LA activation through the BB and the latest RA and LA activation times at 38 ms, 109 ms and 132 ms, respectively, which are within the experimental ranges given by 31±13 ms, 93±17 ms and 116±18 ms registered at the same locations [25]. This is the common approach followed in computational modelling [4,23,50]. Sixth, a torso model containing a carefully adjusted atrial model was constructed from experimental data [30] in order to compute and analyse volumetric electrical propagation, BSPMs, P-waves and the contributions from individual atrial structures. Previous torso models developed to study atrial electrophysiology were modelled using simple spherical models containing the passive volume conductor [2] had a poor geometrical description of the thorax anatomy [40] or included only a subset of organs [51]. Multi-scale atrial models that have included an anatomically detailed torso model, such as [52], have been shown to be able to adequately reproduce clinical data under both healthy and pathological conditions. Our anatomically-detailed atria-torso model was designed with the aim of studying the atria-torso regional correlation and to analyse the individual contribution of each part of the atria in the formation of the P-wave and BSPM, which is an aspect that has not been studied before.

### B. Multi-scale electrophysiology modelling

After coupling the cellular models in 3D, differences in AP morphology were registered when computing and comparing the AP at both levels. These changes, due to the electrical coupling between myocytes, equalize ionic loads and relax the border effects between regions, producing a reduction in the maximum AP amplitude and subtle differences in APD_90_. When considering all action potentials recorded from each atrial region, the APD_90_ value ranges from 178.9±8.2 ms in the PVs to 222.4±4.3 ms in the BB, with a total maximum variability between regions of 56 ms, slightly lower than the variability produced by differences at the cellular level (66 ms between the same regions, see Table 1).

The final multi-scale atrial model produces an activation sequence that matches experimental local activation times. Thus, the latest RA activation time (t = 109 ms) as well as the first and latest LA activations times (t = 38 ms and t = 132 ms respectively) registered in the model are within the experimental ranges of 93±17 ms, 31±13 ms and 116±18 ms, respectively [25].

Using this new multi-scale model, we analysed the influence of both the atrial tissue fibre orientation and the heterogeneity of the torso model on the P-wave characteristics. The effect produced by fibre orientation was also studied at the atrial and torso levels. Our results showed that an isotropic atrial tissue (without considering fibre direction) influences the mean effective velocity and the depolarization pattern, making the depolarization wavefront arrive between 2 and 4 ms earlier at BB and CT but 32ms later at LAA and LPW. These changes are mainly dependent on the degree of alignment between the local direction of fibres in a region and the direction in which the depolarization wavefront enters that region. The transversal direction of the fibres in RAA, TV and LAA therefore slowed down the advance of the wavefront in the anisotropic model. However, in the case of the right inferior LPW, the increase is mainly due to a new wavefront crossing the LFO region and depolarizing the LIPV and inferior LPW faster. The final effects of isotropy on the atrial activation sequence were in agreement with the findings of Roberts et al. [53], who in 1979 experimentally analysed the interdependence between fibre direction, conduction velocity and potential generated by a depolarization wave, concluding that fibre direction has major effects on all of these variables. In the ECG, atrial isotropy produces a reduction in P-wave duration and an increase in its amplitude, mainly due to the above-mentioned changes in the activation atrial pattern. This effect has been experimentally measured by Huo et al. [3], who recently established a causal association between P-wave morphology/duration, histological abnormalities of the atrial myocardium and the major atrial conduction routes.

Our analysis was carried out using a different setup to previous studies [54,55], whose aim was to analyse the combined effect of fibre isotropy and homogeneous atrial conductivity. However, we only used an anisotropy ratio of 1:1, keeping the specific tissue heterogeneity in order to isolate the effect produced by fibre orientation.

The influence of the torso model on the volumetric propagation of the atrial activation and on the morphology of the resulting P-waves and BSPM was also analysed. The first computational torso models considered it to be a finite, homogeneous and isotropic conductor and did not take into account changes produced by tissues and organs [56–58]. The first model that considered different electrical properties of tissues (skin, fat, muscle, bone and vessels) and organs (lungs and heart) was developed by MacLeod et al. in 1991 [30], who used 116 MRIs from a single patient. Subsequent studies [59–61] were able to predict changes in forward-calculated body surface potential maps (BSPMs) caused by variations in tissue conductivities. Recent torso models [41,62] tried to adapt the anatomical form of the torso to different types of patients, aiming to deal with the inverse problem in electrocardiography. The torso model developed in the present work includes all these previous elements: a realistic anatomy derived from an image sequence, the most influential organs with realistic conductivity values and a highly refined area surrounding the atrial myocardium to increase the number of nodes where the extracellular potential is calculated. This new model makes it possible not only to study surface maps, but also volumetric maps, which can help in understanding the influence on P-wave morphology of: atrial fibre direction, electrophysiological tissue heterogeneity, local atrial conductivity and anisotropy, and organ conductivities.

Consequently, our results show that realistic atrial fibre orientation and heterogeneous torso models are essential characteristics of multi-scale models used to simulate human atrial and torso behaviours.

### C. Body Potential Maps and P-wave

In clinical practice it is rather difficult to acquire electro anatomical maps (EAMs) simultaneously to BSPMs, and impossible for healthy subjects. Furthermore, only limited data on BSPM is available in clinical routine. This is due to the relative novelty of BSPM, the lack of consensus in the number and position of leads in experimental BSPM [63] and the difficulty of interpreting such a large amount of information [64]. The enhanced spatial resolution provided by the multiple electrode system provides more detailed information on temporal and spatial distributions of cardiac electrical activity than the standard 12-lead electrocardiography [65–67]. Also, as has already been stated, understanding BSPM during normal cardiac excitation should also serve as the baseline for understanding abnormal cardiac electrical activity and rhythm disorders of the heart [68].

The use of computational modelling techniques and the specific atria-torso multi-scale model developed here may help not only to understand normal atrial excitation and how this sequence physiologically propagates through the torso, but also i) to study the volumetric and superficial effect produced by the excitation of the complete atria and also those produced by individual atrial structures; and ii) to rapidly infer morphological information on the superficial P-waves in any lead.

We have shown that when considering anisotropy, physiological atrial depolarization produces a standard electrical pattern on the BSPM different from that produced when fibre orientation is neglected (Figs 7–9) and comparable to those experimentally measured by [68–71]. Thus, a dipolar distribution characterizes the surface potential maps with negative and positive time integral distributions located in the frontal upper right and lower left quadrants, respectively. This general behaviour is also in agreement with previous computational atria-torso models [2,4,40] although the latter models use a simplified torso anatomy or consider only a limited number of organs.

Additionally, the use of FEM instead of BEM to simulate electrical propagation allowed us to analyse the surface maps (Fig 7b) and to understand how BSPM is formed from the propagation of the electrical wavefront through the torso volume [64]. We analysed the volumetric propagation by means of slices of the torso from multiple axial views (Fig 7c) and studied the spatio-temporal volumetric distribution of the electrical dipole. It was thus possible to determine the part of the atria responsible for the negative and positive time integrals in the frontal view (negatives produced by CT/IB and positives produced by RLW/RAA) and in the rear view (positive time integrals start appearing from t = 70ms mainly dominated by the LSW, LPW and LPV) of the torso surface.

We analysed the P-wave in individual torso leads by means of the root mean square (RMS) value, to simplify the analysis of the spatial and morphological variations of the P-wave and BSPM [72,73]. This method therefore allowed us to extract information on total P-wave morphology and amplitude by observing the RMS distribution map (Fig 8). The expected P-wave morphologies, such as those registered in specific areas of the torso surface with positive and negative single-phase, as well as biphasic or M-shaped complexes, are in agreement with experimental measurements reported by [3,70]. This approach also made it possible to study the individual RMS patterns (Fig 10) and the individual pseudo P-waves (Fig 12) registered on the torso surface. Our RMS study shows that only 9 regions are responsible for almost 90% of the total atrial contribution to the RMS torso surface pattern (Fig 11), while two regions of RA (RLW and RAA) and two of LA (LPW and LPV) are mainly responsible for the total P-wave morphology. However, as there are no previous experimental or computational studies that show how individual atrial structures contribute to the body surface maps it has not been possible to validate our results.

## Conclusions

We consider the major contributions of our study are: 1) a realistic and highly detailed multi-scale atrial-torso model to analyse atrial activation and 2) the possibility of using this model to determine the contribution of different atrial structures to the P-wave and the BSPM distribution in healthy subjects.

This multi-scale atrial-torso model has three main characteristics: 1) electrophysiological heterogeneity due to eight different cellular models with long-term stability in terms of APD_90_ and restitution properties that guarantee the stability of numerical computations; 2) the realistic atrial model has high spatial resolution and is divided into small structures to improve the local control of the histological fibre orientation and electrical properties; 3) a realistic torso model with the highest spatial resolution in the area surrounding the atrium, the most influential tissue conductivities on the generation of body surface and volumetric potentials, and P-wave morphologies.

The simulations carried out using this multi-scale model allowed us to identify the contributions from different parts of the atria and determine their influence on the generation of the P-wave, body surface potential and RMS maps. From the results obtained we concluded that RAA and RLW were the two atrial regions with the biggest influence on the torso frontal surface under normal conditions, due to their size, location and orientation. Furthermore, as P-wave signals in the rear view of the torso surface were mainly produced by LA activation and specifically by the LPW and pulmonary veins, their influence could be analysed individually. The most appropriate locations to register complete P-waves are the centre and the upper right quadrant in the frontal view and the lower left quadrant in the frontal and rear views. Our multi-scale model is an excellent tool to study the effects of pathological substrate from a given region of the atria on the BSPM in order to determine the relationship between pathological substrates and BSPM. It is therefore a promising approach to quantifying the specific effect of pathological regions in the BSPM and to characterising typical scenarios linked to arrhythmic episodes.

## Supporting Information

S1 Fig Body surface RMS maps from the 21 individualized atrial regions. (TIFF)

S2 Fig P-waves produced by the RA structures and registered on precordial and standard leads. For each lead: left panel shows the standard P-wave (continuous black line), the P-wave produced by the RA (dotted black line) and the P-waves produced by individual structures with a contribution higher than 30% of the total P-wave maximum amplitude (colored lines); right panel shows the individual P-waves from RA structures below that 30% (the remaining atrial structures).
(TIFF)

S3 Fig P-waves produced by the LA structures and registered on precordial and standard leads. For each lead: left panel shows the standard P-wave (continuous black line), the P-wave produced by the LA (dotted black line) and the P-waves produced by individual structures with a contribution higher than 30% of the total P-wave maximum amplitude (colored lines); right panel shows the individual P-waves from LA structures below that 30% (the remaining atrial structures).
(TIFF)

S4 Fig P-waves produced by the RA structures and registered on the back of the torso. For each lead: left panel shows the standard P-wave (continuous black line), the P-wave produced by the RA (dotted black line) and the P-waves produced by individual structures with a contribution higher than 30% of the total P-wave maximum amplitude (colored lines); right panel shows the individual P-waves from RA structures below that 30% (the remaining atrial structures).
(TIFF)

S5 Fig P-waves produced by the LA structures and registered on the back of the torso. For each lead: left panel shows the standard P-wave (continuous black line), the P-wave produced by the LA (dotted black line) and the P-waves produced by individual structures with a contribution higher than 30% of the total P-wave maximum amplitude (colored lines); right panel shows the individual P-waves from LA structures below that 30% (the remaining atrial structures).
(TIFF)

S1 Table List of atrial regions and their main electrical properties. (PDF)

S1 Video Atrial activation sequence. Upper left: frontal view; Upper right: rear view; Lower left: inferior view; Lower right: right lateral view.
(AVI)

S2 Video Atrial and torso simulations. Upper left: atrial activation sequence; Upper right: BSPM with isochronal lines; Lower left: transvers plane of the torso model at the V1 and V2 height; Lower right: P-waves registered at the precordial leads V1 to V6.
(AVI)

## References

[1] January CT, Wann LS, Alpert JS, Calkins H, Cigarroa JE, Cleveland JC, et al 2014 AHA/ACC/HRS Guideline for the Management of Patients With Atrial Fibrillation. J Am Coll Cardiol. 2014;64: e1–e76. 10.1016/j.jacc.2014.03.022 24685669

[2] Rodrigo M, Guillem MS, Climent AM, Pedrón-Torrecilla J, Liberos A, Millet J, et al Body surface localization of left and right atrial high-frequency rotors in atrial fibrillation patients: A clinical-computational study. Hear Rhythm. Elsevier; 2014;11: 1584–91. 10.1016/j.hrthm.2014.05.013 PMC4292884 24846374

[3] Huo Y, Mitrofanova L, Orshanskaya V, Holmberg P, Holmqvist F, Platonov PG. P-wave characteristics and histological atrial abnormality. J Electrocardiol. Elsevier Inc.; 2014;47: 275–280. 10.1016/j.jelectrocard.2014.01.011 24602335

[4] Krueger MW, Dorn A, Keller DUJ, Holmqvist F, Carlson J, Platonov PG, et al In-silico modeling of atrial repolarization in normal and atrial fibrillation remodeled state. Med Biol Eng Comput. 2013;51: 1105–1119. 10.1007/s11517-013-1090-1 23864549

[5] Ho SY, Sánchez-Quintana D. The importance of atrial structure and fibers. Clin Anat. 2009;22: 52–63. 10.1002/ca.20634 18470938

[6] Maesen B, Zeemering S, Afonso C, Eckstein J, Burton R a B, Van Hunnik A, et al Rearrangement of atrial bundle architecture and consequent changes in anisotropy of conduction constitute the 3-dimensional substrate for atrial fibrillation. Circ Arrhythmia Electrophysiol. 2013;6: 967–975. 10.1161/CIRCEP.113.000050 23969531

[7] Seemann G, Höper C, Sachse FB, Dössel O, Holden A V, Zhang H. Heterogeneous three-dimensional anatomical and electrophysiological model of human atria. Philos Trans A Math Phys Eng Sci. 2006/06/13 ed. 2006;364: 1465–1481. 10.1098/rsta.2006.1781 16766355

[8] Tobón C, Ruiz-Villa C a., Heidenreich E, Romero L, Hornero F, Saiz J. A Three-Dimensional Human Atrial Model with Fiber Orientation. Electrograms and Arrhythmic Activation Patterns Relationship. PLoS One. 2013;8: e50883 10.1371/journal.pone.0050883 23408928 PMC3569461

[9] Aslanidi OV, Nikolaidou T, Zhao J, Smaill BH, Gilbert SH, Holden A V., et al Application of micro-computed tomography with iodine staining to cardiac imaging, segmentation, and computational model development. IEEE Trans Med Imaging. 2013;32: 8–17. 10.1109/TMI.2012.2209183 22829390 PMC3493467

[10] Aslanidi O V., Butters TD, Ren CX, Ryecroft G, Zhang H. Electrophysiological models for the heterogeneous canine atria: Computational platform for studying rapid atrial arrhythmias. Proc Annu Int Conf IEEE Eng Med Biol Soc EMBS. 2011;2011: 1693–1696. 10.1109/IEMBS.2011.6090486 PMC3403810 22254651

[11] Maleckar MM, Greenstein JL, Giles WR, Trayanova N a. K+ current changes account for the rate dependence of the action potential in the human atrial myocyte. Am J Physiol Heart Circ Physiol. 2009/07/28 ed. 2009;297: H1398–H1410. 10.1152/ajpheart.00411.2009 19633207 PMC2770776

[12] Cabrera JA, Ho SY, Climent V, Sánchez-Quintana D. The architecture of the left lateral atrial wall: A particular anatomic region with implications for ablation of atrial fibrillation. Eur Heart J. 2008/02/05 ed. 2008;29: 356–362. 10.1093/eurheartj/ehm606 18245120

[13] Ho SY, Cabrera JA, Sanchez-Quintana D. Left atrial anatomy revisited. Circ Arrhythmia Electrophysiol. 2012;5: 220–228. 10.1161/CIRCEP.111.962720 22334429

[14] Sánchez-Quintana D, López-Mínguez JR, Pizarro G, Murillo M, Cabrera JA. Triggers and anatomical substrates in the genesis and perpetuation of atrial fibrillation. Curr Cardiol Rev. 2012;8: 310–26. 10.2174/157340312803760721 22920484 PMC3492815

[15] Sánchez-Quintana D, Pizarro G, López-Mínguez JR, Ho SY, Cabrera JA. Standardized review of atrial anatomy for cardiac electrophysiologists. J Cardiovasc Transl Res. 2013;6: 124–144. 10.1007/s12265-013-9447-2 23389853

[16] Hansson a., Holm M, Blomström P, Johansson R, Lührs C, Brandt J, et al Right atrial free wall conduction velocity and degree of anisotropy in patients with stable sinus rhythm studied during open heart surgery. Eur Heart J. 1998/03/31 ed. 1998;19: 293–300. 10.1053/euhj.1997.0742 9519324

[17] Ho SY, McCarthy KP. Anatomy of the left atrium for interventional electrophysiologists. PACE—Pacing Clin Electrophysiol. 2010;33: 620–627. 10.1111/j.1540-8159.2009.02659.x 20025713

[18] Feng J, Yue L, Wang Z, Nattel S. Ionic mechanisms of regional action potential heterogeneity in the canine right atrium. Circ Res. 1998;83: 541–551. 10.1161/01.RES.83.5.541 9734477

[19] Li D, Zhang L, Kneller J, Nattel S. Potential ionic mechanism for repolarization differences between canine right and left atrium. Circ Res. 2001;88: 1168–1175. 10.1161/hh1101.091266 11397783

[20] Cha TJ, Ehrlich JR, Zhang L, Chartier D, Leung TK, Nattel S. Atrial tachycardia remodeling of pulmonary vein cardiomyocytes: Comparison with left atrium and potential relation to arrhythmogenesis. Circulation. 2005;111: 728–735. 10.1161/01.CIR.0000155240.05251.D0 15699259

[21] Wang ZG, Pelletier LC, Talajic M, Nattel S. Effects of flecainide and quinidine on human atrial action potentials. Role of rate-dependence and comparison with guinea pig, rabbit, and dog tissues. Circulation. 1990;82: 274–283. 10.1161/01.CIR.82.1.274 2114235

[22] Wang Z, Fermini B, Nattel S. Sustained depolarization-induced outward current in human atrial myocytes. Evidence for a novel delayed rectifier K+ current similar to Kv1.5 cloned channel currents. Circ Res. 1993;73: 1061–1076. 10.1161/01.RES.73.6.1061 8222078

[23] Colman M. Mechanisms of Atrial Arrhythmias [Internet]. Nature reviews. Cardiology. Springer International Publishing. 2014 10.1007/978-3-319-01643-6

[24] Dobrev D, Graf E, Wettwer E, Himmel HM, Hála O, Doerfel C, et al Molecular basis of downregulation of G-protein-coupled inward rectifying K(+) current (I(K,ACh) in chronic human atrial fibrillation: decrease in GIRK4 mRNA correlates with reduced I(K,ACh) and muscarinic receptor-mediated shortening of action potentials. Circulation. 2001;104: 2551–2557. 10.1161/hc4601.099466 11714649

[25] Lemery R, Birnie D, Tang ASL, Green M, Gollob M, Hendry M, et al Normal atrial activation and voltage during sinus rhythm in the human heart: An endocardial and epicardial mapping study in patients with a history of atrial fibrillation. J Cardiovasc Electrophysiol. 2007/03/31 ed. 2007;18: 402–408. 10.1111/j.1540-8167.2007.00762.x 17394455

[26] Saffitz JE, Kanter HL, Green KG, Tolley TK, Beyer EC. Tissue-specific determinants of anisotropic conduction velocity in canine atrial and ventricular myocardium. Circ Res. 1994;74: 1065–1070. 10.1161/01.RES.74.6.1065 8187276

[27] Spach MS, Dolber PC, Heidlage JF. Influence of the passive anisotropic properties on directional differences in propagation following modification of the sodium conductance in human atrial muscle. A model of reentry based on anisotropic discontinuous propagation. Circ Res. 1988;62: 811–832. 10.1161/01.RES.62.4.811 2450697

[28] Fedorov V, Glukhov AV, Chang R, Kostecki G, Aferol H, Hucker WJ, et al Optical mapping of the isolated coronary-perfused human sinus node. J Am Coll Cardiol. 2010;56: 1386–1394. 10.1016/j.jacc.2010.03.098 20946995 PMC3008584

[29] Kojodjojo P, Kanagaratnam P, Markides V, Davies W, Peters N. Age-Related Changes in Human Left and Right Atrial Conduction. J Cardiovasc Electrophysiol. 2006; 10–15. 10.1111/j.1540-8167.2005.00293.x 16533247

[30] MacLeod RS, Johnson CR, Ershler PR. Construction of an Inhomogeneous Model of the Human Torso for Use in Computational Electrocardiography. IEEE Engineering in Medicine and Biology Society 13th Annual International Conference. IEEE Press; 1991. p. pages 688–689.

[31] Cibc. Seg3D: Volumetric Image Segmentation and Visualization. Scientific Computing and Imaging Institute (SCI), Download from: http://www.seg3d.org [Internet]. 2013. Available: http://www.seg3d.org

[32] Si H, Gärtner K. Meshing piecewise linear complexes by constrained delaunay tetrahedralizations [Internet]. Proceedings of the 14th International Meshing Roundtable, IMR 2005. 2005. 10.1007/3-540-29090-7-9

[33] Bradley CP, Pullan a J, Hunter PJ. Effects of material properties and geometry on electrocardiographic forward simulations. Ann Biomed Eng. 2000;28: 721–741. 11016411 10.1114/1.1289467

[34] Bressler SL, Ding M. Wiley Encyclopedia of Biomedical Engineering : Event-Related Potentials Psychiatry research. John Wiley & Sons, Inc.; 2006 pp. 1–8.

[35] Poletti E. Fiorin D. G E, Ruggeri a. World Congress on Medical Physics and Biomedical Engineering, September 7–12, 2009, Munich, Germany. In: Dössel O, Schlegel WC, editors. Springer Berlin Heidelberg; 2009 pp. 137–140.

[36] Gabriel S, Lau RW, Gabriel C. The dielectric properties of biological tissues: II. Measurements in the frequency range 10 Hz to 20 GHz. Phys Med Biol. 1996;41: 2251–2269. 10.1088/0031-9155/41/11/002 8938025

[37] Klepfer RN, Johnson CR, Macleod RS. The effects of inhomogeneities and anisotropies on electrocardiographic fields: A 3-D finite-element study. IEEE Trans Biomed Eng. 1997/08/01 ed. 1997;44: 706–719. 10.1109/10.605427 9254984

[38] Malmivuo J, Plonsey R. Bioelectromagnetism: Principles and Applications of Bioletric and Biomagnetic Fields. Plonsey R, editor. New York : Oxford University Press; 1995.

[39] Heidenreich EA, Ferrero JM, Doblaré M, Rodríguez JF. Adaptive macro finite elements for the numerical solution of monodomain equations in cardiac electrophysiology. Ann Biomed Eng. 2010;38: 2331–2345. 10.1007/s10439-010-9997-2 20238165

[40] Aslanidi O, Colman MA, Stott J, Dobrzynski H, Boyett MR, Holden A V, et al 3D virtual human atria: A computational platform for studying clinical atrial fibrillation. Prog Biophys Mol Biol. 2011/07/19 ed. 2011;107: 156–168. 10.1016/j.pbiomolbio.2011.06.011 21762716 PMC3211061

[41] Krueger MW, Seemann G, Rhode K, Keller DUJ, Schilling C, Arujuna A, et al Personalization of atrial anatomy and electrophysiology as a basis for clinical modeling of radio-frequency ablation of atrial fibrillation. IEEE Trans Med Imaging. 2013;32: 73–84. 10.1109/TMI.2012.2201948 22665507

[42] Krogh-Madsen T, Abbott GW, Christini DJ. Effects of electrical and structural remodeling on atrial fibrillation maintenance: A simulation study. PLoS Comput Biol. 2012;8 Available: http://www.scopus.com/inward/record.url?eid=2-s2.0-84861143547&partnerID=40&md5=ecea4d4be5398af84a285e4480042f55 10.1371/journal.pcbi.1002390 PMC3285569 22383869

[43] Dössel O, Krueger MW, Weber FM, Wilhelms M, Seemann G. Computational modeling of the human atrial anatomy and electrophysiology. Med Biol Eng Comput. 2012;50: 773–799. 10.1007/s11517-012-0924-6 22718317

[44] Aslanidi O, Al-Owais M, Benson AP, Colman M, Garratt CJ, Gilbert SH, et al Virtual tissue engineering of the human atrium: Modelling pharmacological actions on atrial arrhythmogenesis. Eur J Pharm Sci. Elsevier B.V.; 2012;46: 209–221. 10.1016/j.ejps.2011.08.014 21888968

[45] Burdumy M, Luik A, Neher P, Hanna R, Krueger MW, Schilling C, et al Comparing measured and simulated wave directions in the left atrium a workflow for model personalization and validation. Biomed Tech. 2012;57: 79–87. 10.1515/bmt-2011-0059 22505490

[46] Lamata P, Roy I, Blazevic B, Crozier A, Land S, Niederer S a., et al Quality metrics for high order meshes: Analysis of the mechanical simulation of the heart beat. IEEE Trans Med Imaging. 2013;32: 130–138. 10.1109/TMI.2012.2231094 23221814

[47] Cabrera JA, Sánchez-Quintana D. Cardiac anatomy: what the electrophysiologist needs to know. Heart. 2013;99: 417–31. 10.1136/heartjnl-2011-301154 23355600

[48] Nygren a, Fiset C, Firek L, Clark JW, Lindblad DS, Clark RB, et al Mathematical model of an adult human atrial cell: the role of K+ currents in repolarization. Circ Res. 1998/01/24 ed. 1998;82: 63–81. 10.1161/01.RES.82.1.63 9440706

[49] Wilhelms M, Hettmann H, Maleckar MM, Koivumäki JT, Dössel O, Seemann G. Benchmarking electrophysiological models of human atrial myocytes. Front Physiol. 2013;3 1: 487 10.3389/fphys.2012.00487 23316167 PMC3539682

[50] Deng D, Gong Y, Shou G, Jiao P, Zhang H, Ye X, et al Simulation of biatrial conduction via different pathways during sinus rhythm with a detailed human atrial model. J Zhejiang Univ Sci B. 2012;13: 676–694. 10.1631/jzus.B1100339 22949359 PMC3437366

[51] Boulakia M, Cazeau S, Fernández M a., Gerbeau JF, Zemzemi N. Mathematical modeling of electrocardiograms: A numerical study. Ann Biomed Eng. 2009/12/25 ed. 2010;38: 1071–1097. 10.1007/s10439-009-9873-0 20033779

[52] Krueger MW, Rhode K, Weber FM, Keller DUJ, Caulfield D, Seemann G, et al Modellbildung und Simulation. Biomed Tech Eng. 2010;55: 1–237. 10.1515/bmt.2010.712

[53] Roberts DE, Hersh LT, Scher a M. Influence of cardiac fiber orientation on wavefront voltage, conduction velocity, and tissue resistivity in the dog. Circ Res. 1979;44: 701–712. 10.1161/01.RES.44.5.701 428066

[54] Krüger M. Personalized Multi-Scale Modeling of the Atria. Karlsruher Institut für Technologie (KIT). 2012.

[55] Zhao J, Butters TD, Zhang H, Pullan AJ, LeGrice IJ, Sands GB, et al An image-based model of atrial muscular architecture effects of structural anisotropy on electrical activation. Circ Arrhythmia Electrophysiol. 2012/03/17 ed. 2012;5: 361–370. 10.1161/CIRCEP.111.967950 22423141

[56] Rudy Y, Plonsey R. A comparison of volume conductor and source geometry effects on body surface and epicardial potentials. Circ Res. 1980/02/01 ed. 1980;46: 283–291. 10.1161/01.RES.46.2.283 6444278

[57] Abboud S, Berenfeld O, Sadeh D. Simulation of high-resolution QRS complex using a ventricular model with a fractal conduction system. Effects of ischemia on high-frequency QRS potentials. Circ Res. 1991;68: 1751–1760. 10.1161/01.RES.68.6.1751 2036723

[58] Leon LJ, Horacek BM. Computer-Model of Excitation and Recovery in the Anisotropic Myocardium .3. Arrhythmogenic Conditions in the Simplified Left-Ventricle. J Electrocardiol. 1991/01/01 ed. 1991;24: 33–41. Available: http://www.ncbi.nlm.nih.gov/pubmed/2056265 2056266 10.1016/0022-0736(91)90079-2

[59] Weber FM, Keller DUJ, Bauer S, Seemann G, Lorenz C, Dössel O. Predicting tissue conductivity influences on body surface potentials-An efficient approach based on principal component analysis. IEEE Trans Biomed Eng. 2010/11/03 ed. 2011;58: 265–273. 10.1109/TBME.2010.2090151 21041150

[60] Keller DUJ, Weber FM, Seemann G, Dössel O. Ranking the influence of tissue conductivities on forward-calculated ecgs. IEEE Trans Biomed Eng. 2010/07/28 ed. 2010;57: 1568–1576. 10.1109/TBME.2010.2046485 20659824

[61] Van Dam PM, van Oosterom A. Volume conductor effects involved in the genesis of the P wave. Europace. 2005;7: S30–S38. 10.1016/j.eupc.2005.03.013 16102501

[62] Lenkova J, Svehlikova J, Tysler M. Individualized model of torso surface for the inverse problem of electrocardiology. J Electrocardiol. 2012/03/10 ed. Elsevier Inc.; 2012;45: 231–236. 10.1016/j.jelectrocard.2012.01.006 22402335

[63] Guillem M, Bollmann A, Climent AM, Husser D, Millet-Roig J, Castells F. How many leads are necessary for a reliable reconstruction of surface potentials during atrial fibrillation? IEEE Trans Inf Technol Biomed. 2009/05/09 ed. 2009;13: 330–340. 10.1109/TITB.2008.2011894 19423429

[64] Van der Graaf AWM, Bhagirath P, Ramanna H, van Driel VJHM, de Hooge J, de Groot NMS, et al Noninvasive Imaging of Cardiac Excitation: Current Status and Future Perspective. Ann Noninvasive Electrocardiol. 2014; n/a–n/a. 10.1111/anec.12140 PMC6932091 24620843

[65] Zarychta P, Smith FE, King ST, Haigh a J, Klinge a, Zheng D, et al Body Surface Potential Mapping for Detection of Myocardial Infarct Sites. 2007; 181–184.

[66] Fereniec M, Stix G, Kania M, Mroczka T, Janusek D, Maniewski R. Risk assessment of ventricular arrhythmia using new parameters based on high resolution body surface potential mapping. Med Sci Monit. 2011;17: MT26–T33. 10.12659/MSM.881436 21358612 PMC3524725

[67] Safdarian N, Dabanloo NJ, Matini SA, Nasrabadi AM. Rule-based Method for Extent and Localization of Myocardial Infarction by Extracted Features of ECG Signals using Body Surface Potential Map Data. J Med Signals Sens. 2013;3: 129–138. 24672761 PMC3959003

[68] Ramanathan C, Jia P, Ghanem R, Ryu K, Rudy Y. Activation and repolarization of the normal human heart under complete physiological conditions. Proc Natl Acad Sci U S A. 2006;103: 6309–6314. 10.1073/pnas.0601533103 16606830 PMC1458874

[69] SippensGroenewegen A, Peeters HA, Jessurun ER, Linnenbank AC, Robles de Medina EO, Lesh MD, et al Body surface mapping during pacing at multiple sites in the human atrium: P-wave morphology of ectopic right atrial activation. Circulation. 1998;97: 369–380. 10.1161/01.CIR.97.4.369 9468211

[70] Lian J, Li G, Cheng J, Avitall B, He B. Body surface Laplacian mapping of atrial depolarization in healthy human subjects. Med Biol Eng Comput. 2002;40: 650–659. 10.1007/BF02345304 12507316

[71] Kozlíková K. P-wave body surface isointegral maps in children and in young adults. Physiol Res. 2007;56 Suppl 1: S123–S128. Available: http://www.biomed.cas.cz/physiolres/pdf/2007/56_S123.pdf 10.33549/physiolres.931310 17552886

[72] Gozolits S, Fischer G, Berger T, Hanser F, Abou-Harb M, Tilg B, et al Global P Wave Duration on the 65-Lead ECG: Single-Site and Dual-Site Pacing in the Structurally Normal Human Atrium. J Cardiovasc Electrophysiol. 2002;13: 1240–1245. 10.1046/j.1540-8167.2002.01240.x 12521340

[73] Lux RL, Sower CT, Allen N, Etheridge SP, Tristani-Firouzi M, Saarel E V. The application of root mean square electrocardiography (RMS ECG) for the detection of acquired and congenital long QT syndrome. PLoS One. 2014;9: 1–9. 10.1371/journal.pone.0085689 PMC3893255 24454918

