# Supplementary figures and images for: Detailed Anatomical and Electrophysiological Models of Human Atria and Torso for the Simulation of Atrial Activation
Source: PLoS One. 2015 Nov 2;10(11):e0141573. doi: 10.1371/journal.pone.0141573 (PMC4629897; doi:10.1371/journal.pone.0141573)

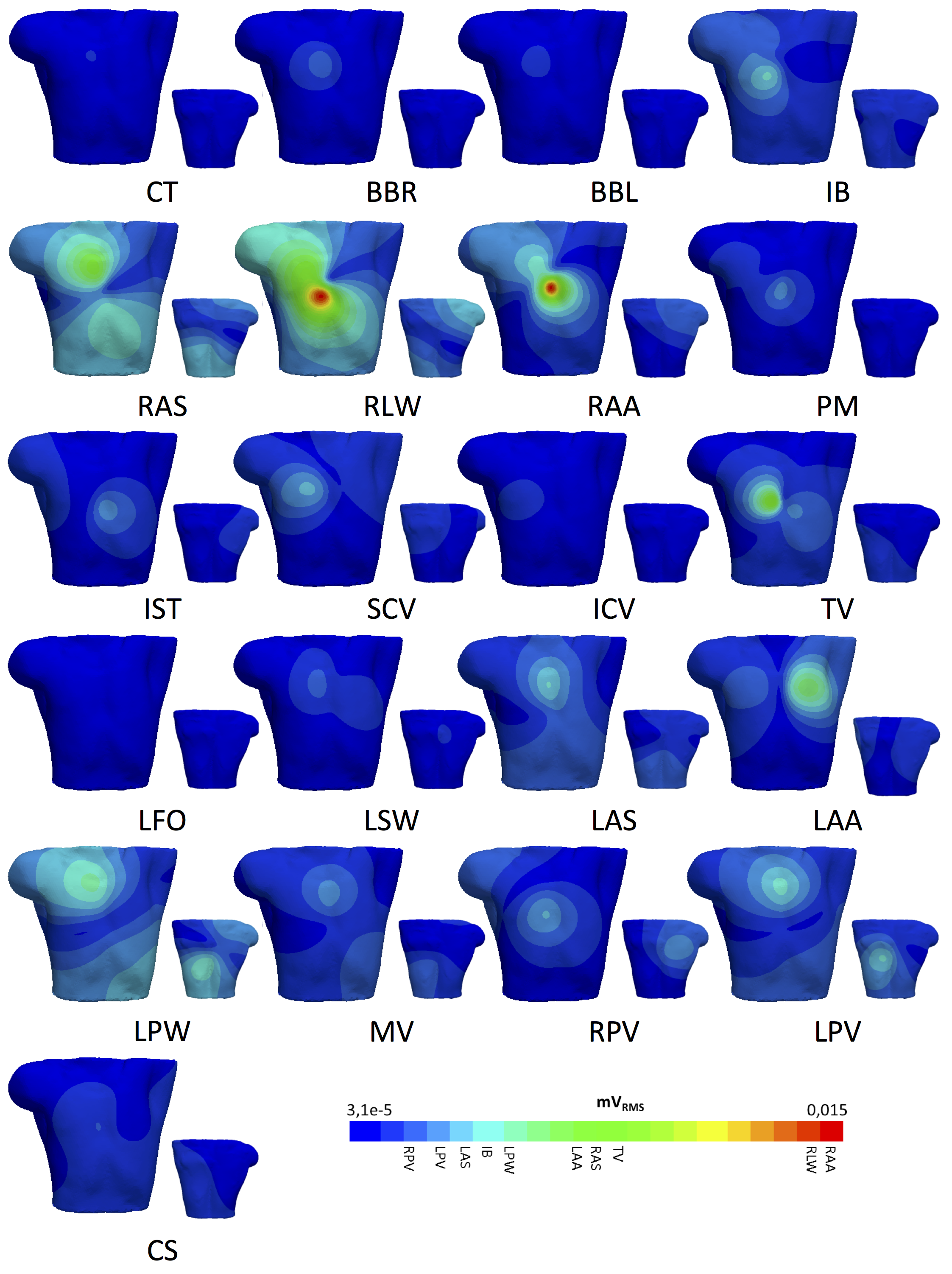

Supplement: S1 Fig — (TIFF) [file pone.0141573.s001.tiff]

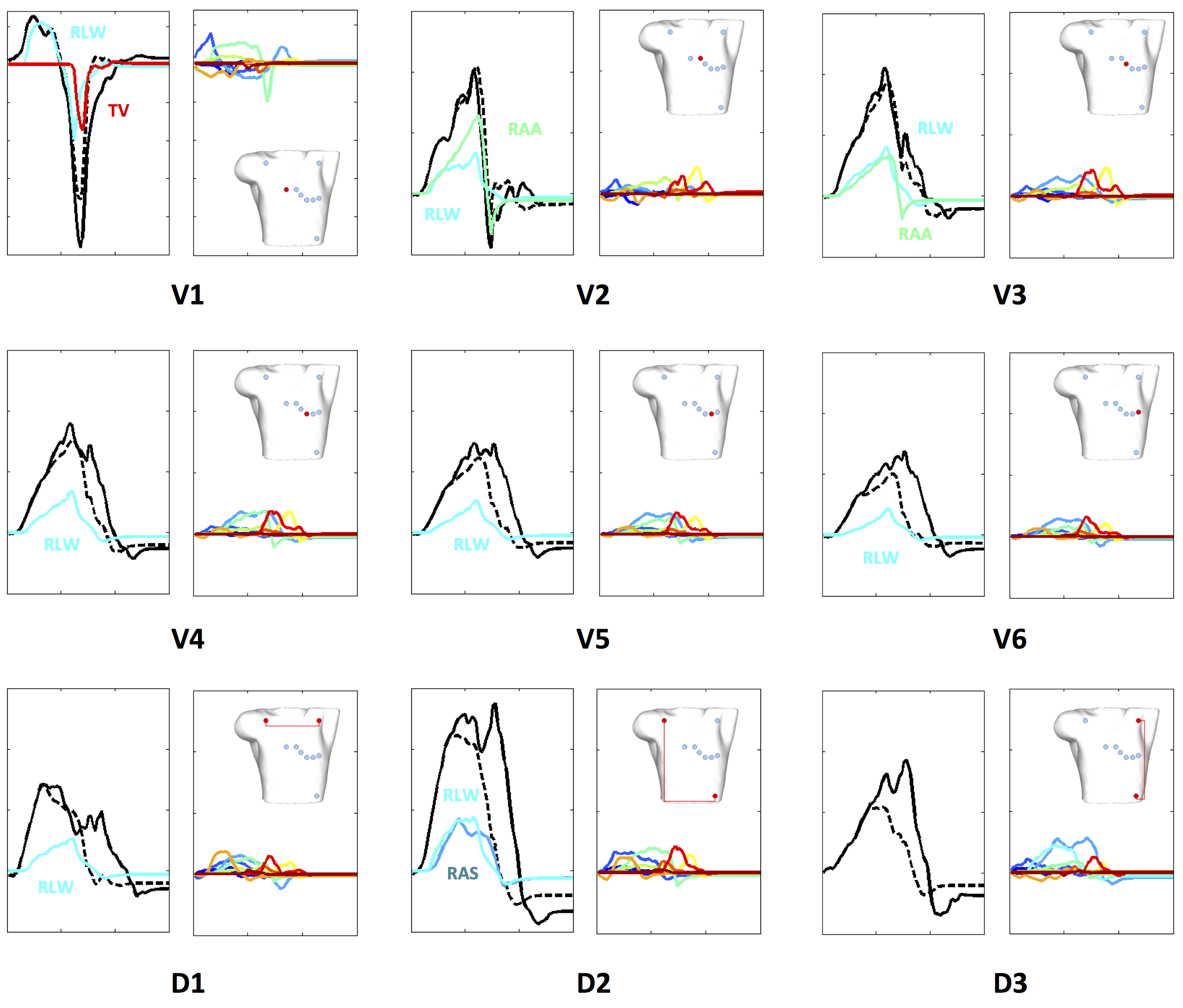

Supplement: S2 Fig — For each lead: left panel shows the standard P-wave (continuous black line), the P-wave produced by the RA (dotted black line) and the P-waves produced by individual structures with a contribution higher than 30% of the total P-wave maximum amplitude (colored lines); right panel shows the individual P-waves from RA structures below that 30% (the remaining atrial structures). (TIFF) [file pone.0141573.s002.tiff]

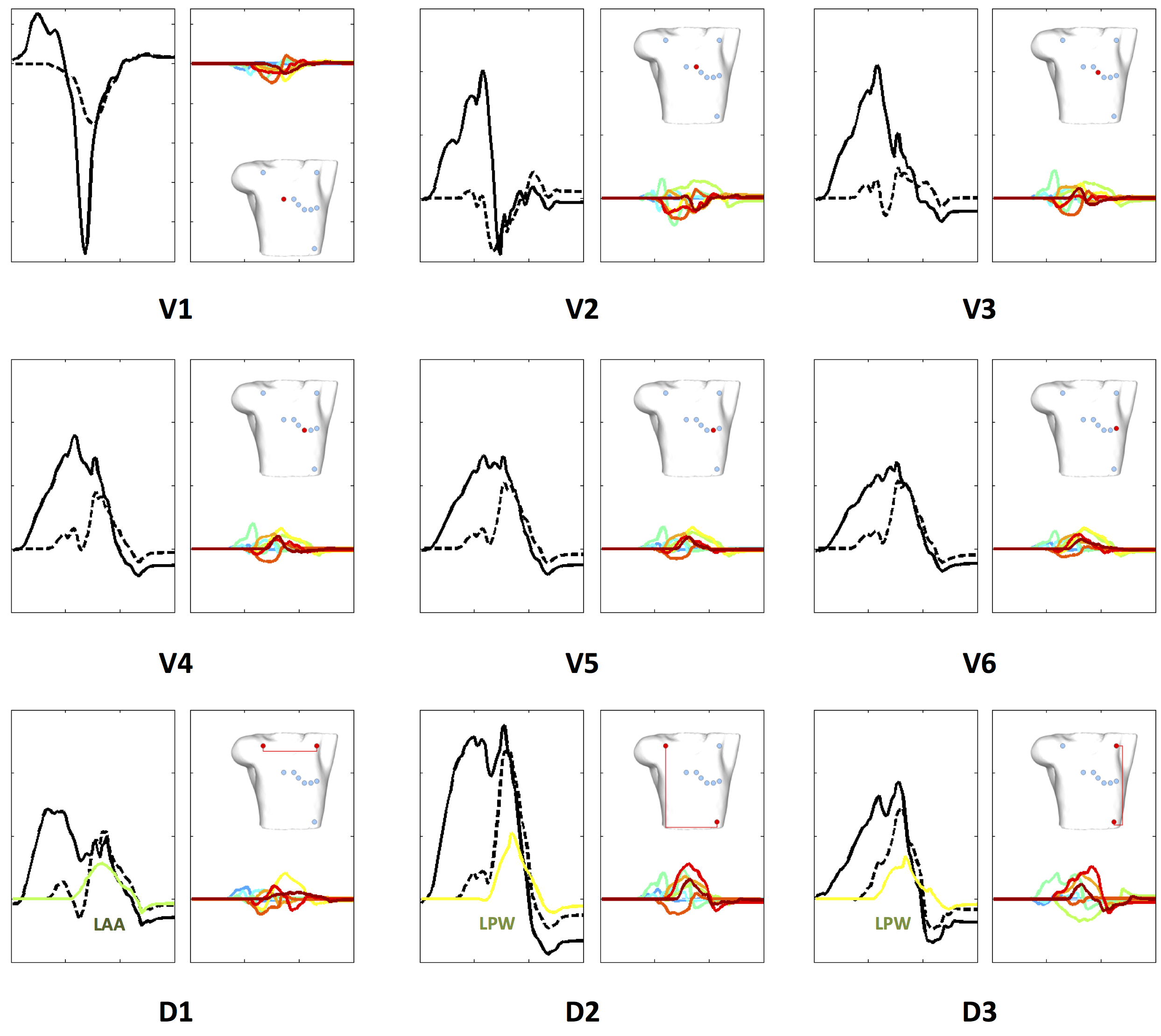

Supplement: S3 Fig — For each lead: left panel shows the standard P-wave (continuous black line), the P-wave produced by the LA (dotted black line) and the P-waves produced by individual structures with a contribution higher than 30% of the total P-wave maximum amplitude (colored lines); right panel shows the individual P-waves from LA structures below that 30% (the remaining atrial structures). (TIFF) [file pone.0141573.s003.tiff]

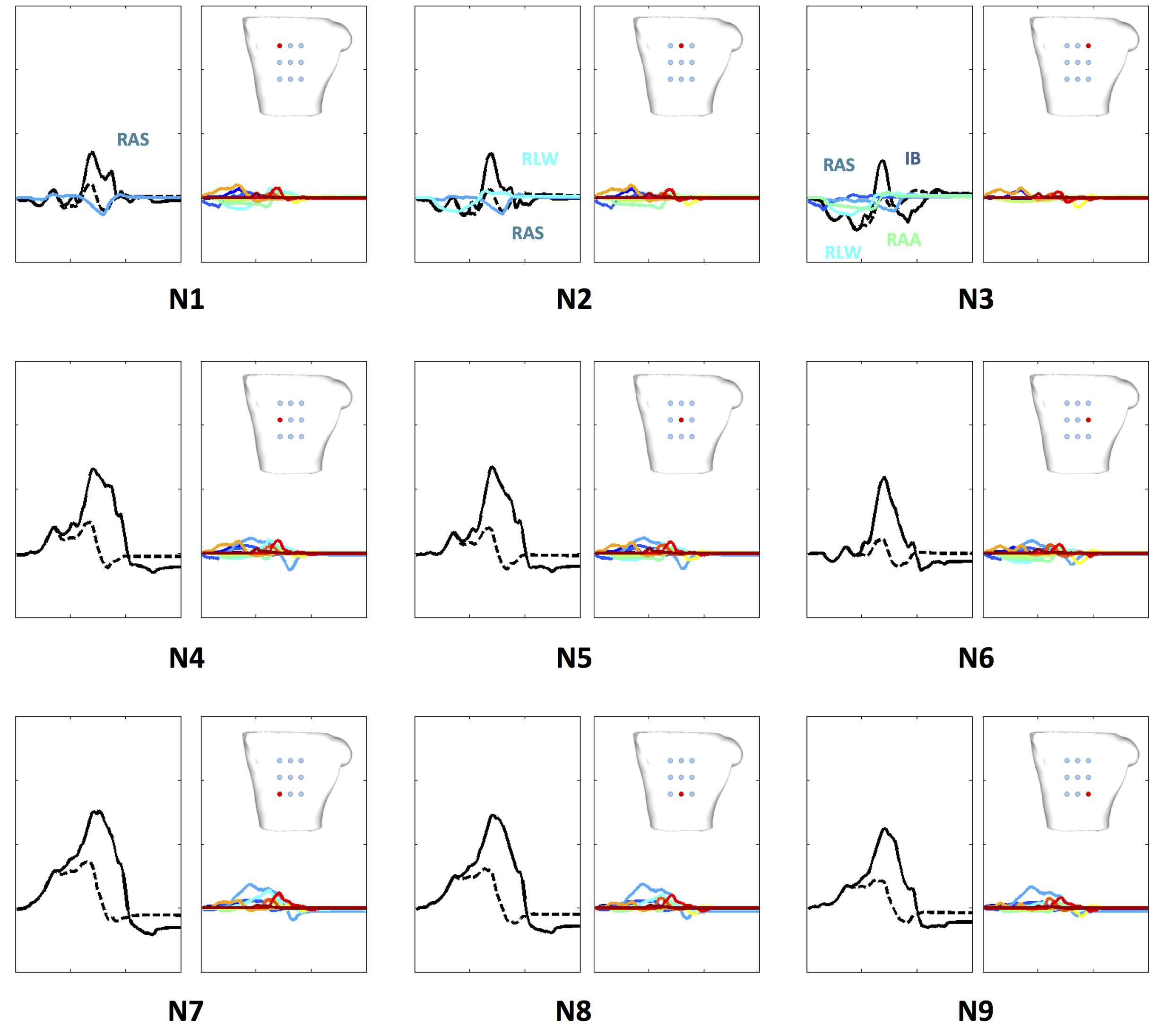

Supplement: S4 Fig — For each lead: left panel shows the standard P-wave (continuous black line), the P-wave produced by the RA (dotted black line) and the P-waves produced by individual structures with a contribution higher than 30% of the total P-wave maximum amplitude (colored lines); right panel shows the individual P-waves from RA structures below that 30% (the remaining atrial structures). (TIFF) [file pone.0141573.s004.tiff]

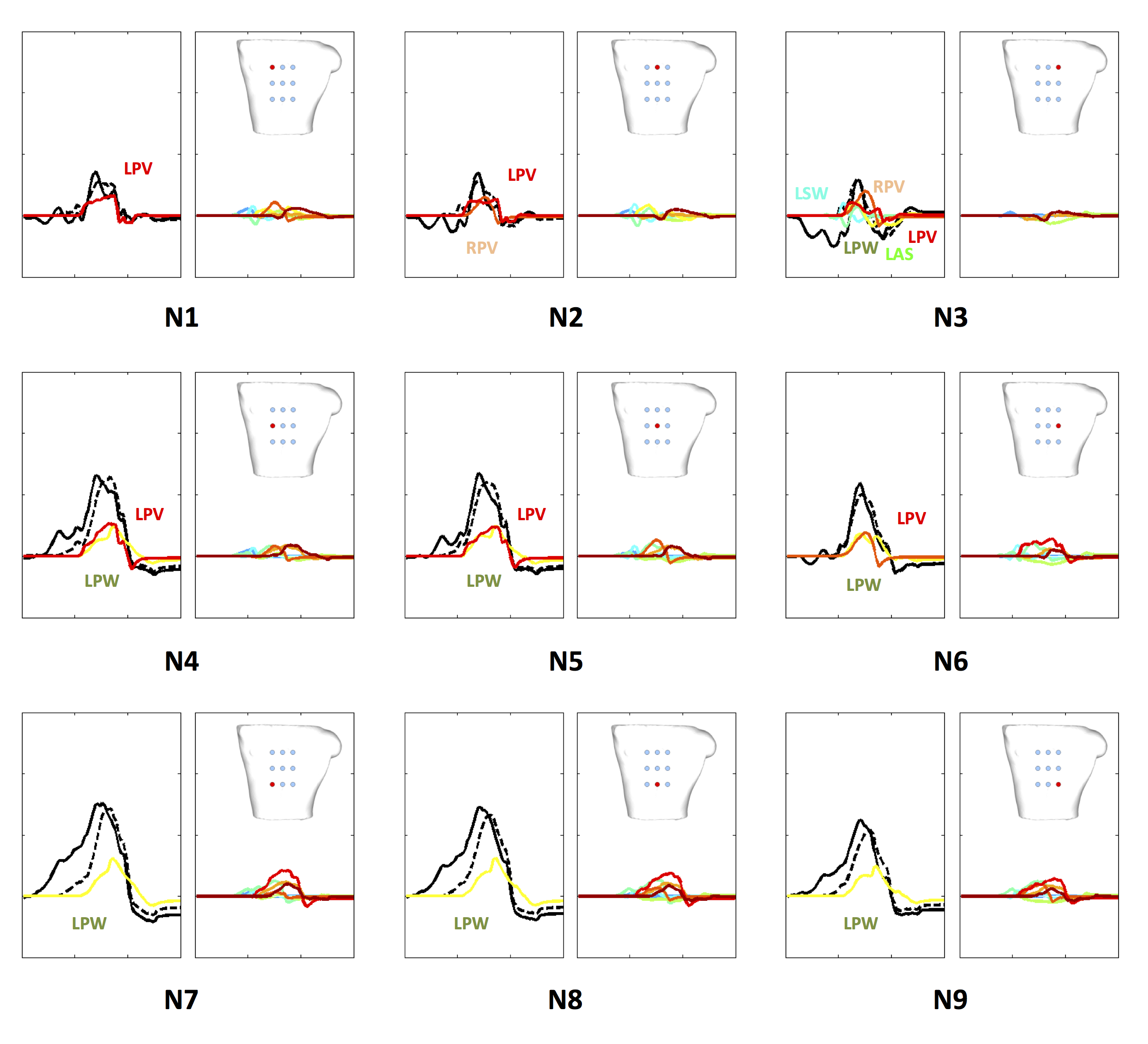

Supplement: S5 Fig — For each lead: left panel shows the standard P-wave (continuous black line), the P-wave produced by the LA (dotted black line) and the P-waves produced by individual structures with a contribution higher than 30% of the total P-wave maximum amplitude (colored lines); right panel shows the individual P-waves from LA structures below that 30% (the remaining atrial structures). (TIFF) [file pone.0141573.s005.tiff]
