# Supplementary material for: Detailed Anatomical and Electrophysiological Models of Human Atria and Torso for the Simulation of Atrial Activation
Source: PLoS One. 2015 Nov 2;10(11):e0141573. doi: 10.1371/journal.pone.0141573 (PMC4629897; doi:10.1371/journal.pone.0141573)
Supplement: S1 Table — (PDF) [file pone.0141573.s006.pdf]

S1 Table: List of atrial regions and their main electrical properties

Nº	REGION	PROPERTY	VALUE	FIBRE DIRECTION	
1	SAN	Nº ELE	659	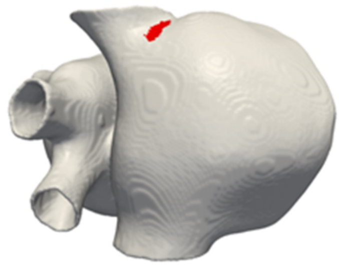	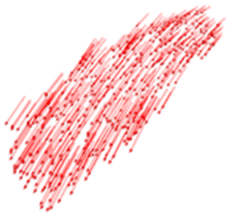
		Sub-regions	–		
		V (mm^3)	17.8		
		MODEL	RA		
		MATERIAL	SAN		
		CV_L	27.7		
		CV_T	27.7		
		$LAT_{INI\ (ms)}$	0		
		$LAT_{END\ (ms)}$	3		
2	CT	Nº ELE	5867	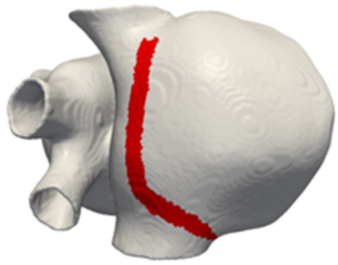	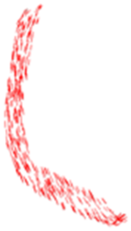
		Sub-regions	–		
		V (mm^3)	158.4		
		MODEL	CT		
		MATERIAL	CT		
		CV_L	100.0		
		CV_T	38.9		
		$LAT_{INI\ (ms)}$	3		
		$LAT_{END\ (ms)}$	60		
3	BBR BBL	Nº ELE	15227	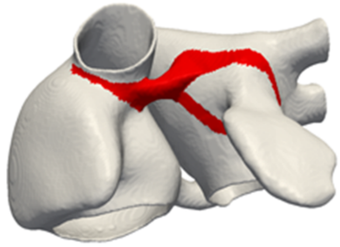	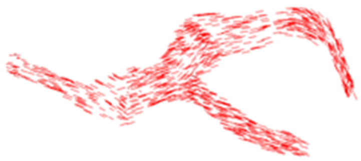
		Sub-regions	6		
		V (mm^3)	411.1		
		MODEL	BB		
		MATERIAL	BB		
		CV_L	116.7		
		CV_T	46.5		
		$LAT_{INI\ (ms)}$	3		
		$LAT_{END\ (ms)}$	72		
	BB (Insulating)	Nº ELE	4430	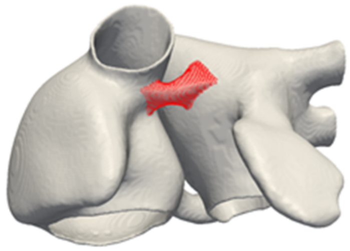	NO FIBRES DIRECTION
		Sub-regions	2		
		V (mm^3)	119.6		
		MODEL	–		
		MATERIAL	–		
		CV_L	0		
		CV_T	0		
		$LAT_{INI\ (ms)}$	0		
		$LAT_{END\ (ms)}$	0		
4	IB	Nº ELE	25732	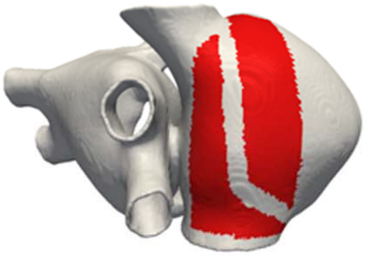	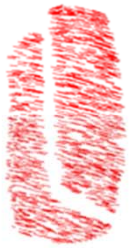
		Sub-regions	–		
		V (mm^3)	694.8		
		MODEL	RA		
		MATERIAL	RA		
		CV_L	63.3		
		CV_T	36.6		
		$LAT_{INI\ (ms)}$	3		
		$LAT_{END\ (ms)}$	60		
5	RAS	Nº ELE	48977	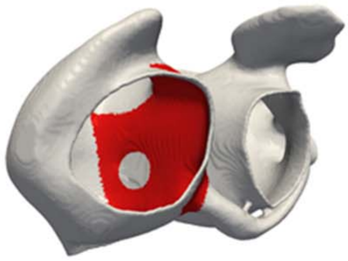	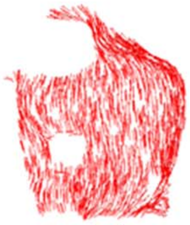
		Sub-regions	–		
		V (mm^3)	1322.4		
		MODEL	RA		
		MATERIAL	RA		
		CV_L	63.3		
		CV_T	36.6		
		$LAT_{INI\ (ms)}$	20		
		$LAT_{END\ (ms)}$	95		

Nº	REGION	PROPERTY	VALUE	FIBRE DIRECTION	
6	RLW	Nº ELE	40932	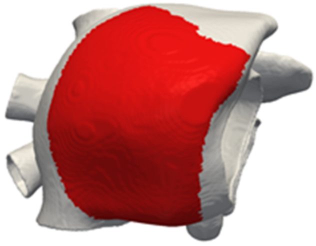	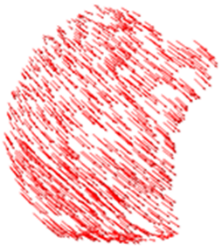
		Sub-regions	–		
		V (mm^3)	1105.2		
		MODEL	RA		
		MATERIAL	RA		
		CV_L	63.3		
		CV_T	36.6		
		$LAT_{INI\ (ms)}$	18		
		$LAT_{END\ (ms)}$	85		
7	RAA	Nº ELE	27740	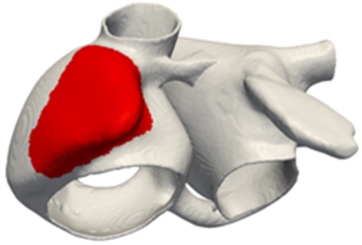	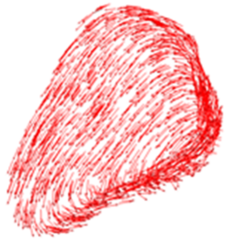
		Sub-regions	–		
		V (mm^3)	749.0		
		MODEL	RAA		
		MATERIAL	RA		
		CV_L	63.3		
		CV_T	36.5		
		$LAT_{INI\ (ms)}$	20		
		$LAT_{END\ (ms)}$	75		
8	PM	Nº ELE	15259	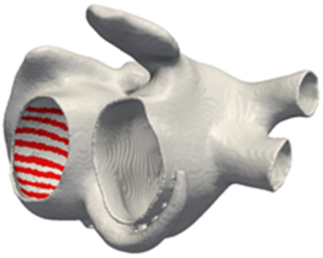	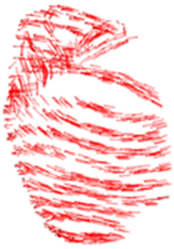
		Sub-regions	10		
		V (mm^3)	412.0		
		MODEL	PM		
		MATERIAL	PM		
		CV_L	115.4		
		CV_T	46.3		
		$LAT_{INI\ (ms)}$	15		
		$LAT_{END\ (ms)}$	75		
9	IST	Nº ELE	9023	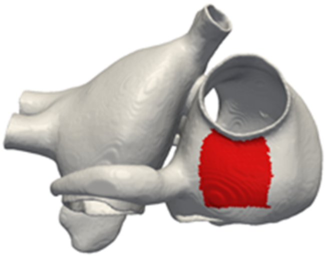	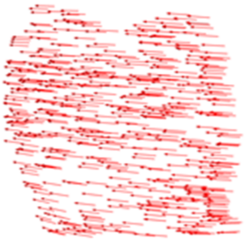
		Sub-regions	–		
		V (mm^3)	243.6		
		MODEL	RA		
		MATERIAL	IST		
		CV_L	76.6		
		CV_T	76.6		
		$LAT_{INI\ (ms)}$	75		
		$LAT_{END\ (ms)}$	96		
10	SCV	Nº ELE	19121	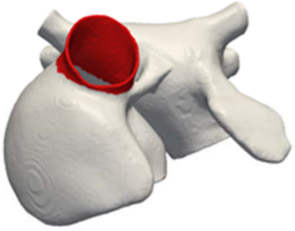	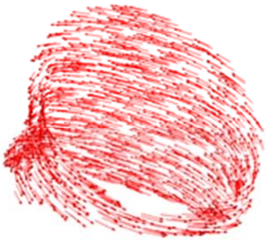
		Sub-regions	–		
		V (mm^3)	516.3		
		MODEL	RA		
		MATERIAL	RA		
		CV_L	63.3		
		CV_T	36.6		
		$LAT_{INI\ (ms)}$	3		
		$LAT_{END\ (ms)}$	52		
11	ICV	Nº ELE	5231	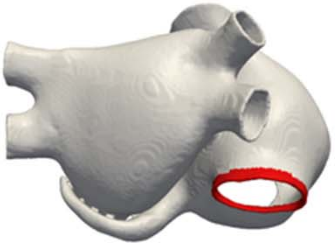	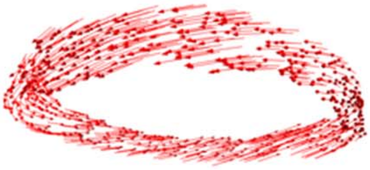
		Sub-regions	–		
		V (mm^3)	141.2		
		MODEL	RA		
		MATERIAL	RA		
		CV_L	63.3		
		CV_T	36.6		
		$LAT_{INI\ (ms)}$	55		
		$LAT_{END\ (ms)}$	92		

Nº	REGION	PROPERTY	VALUE	FIBRE DIRECTION	
12	TV	Nº ELE	15194	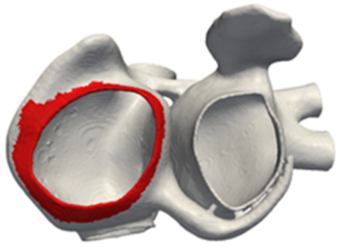	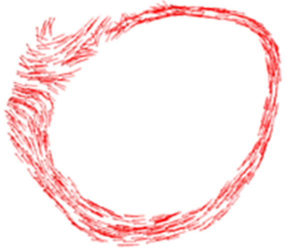
		Sub-regions	–		
		V (mm³)	410.2		
		MODEL	TV		
		MATERIAL	RA		
		CV_L	63.3		
		CV_T	36.5		
		LAT_{INI} (ms)	64		
		LAT_{END} (ms)	109		
13	LFO	Nº ELE	4654	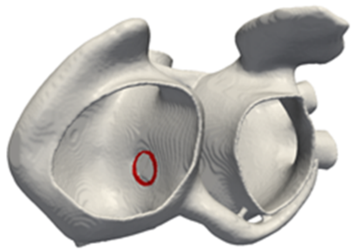	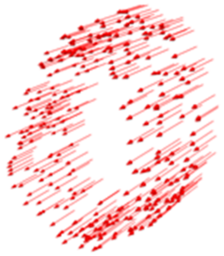
		Sub-regions	–		
		V (mm³)	125.7		
		MODEL	RA		
		MATERIAL	LFO		
		CV_L	99.1		
		CV_T	38.6		
		LAT_{INI} (ms)	48		
		LAT_{END} (ms)	80		
	FO (Insulating)	Nº ELE	6357	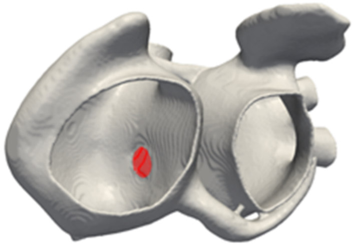	NO FIBRES DIRECTION
		Sub-regions	–		
		V (mm³)	171.6		
		MODEL	–		
		MATERIAL	–		
		CV_L	0.0		
		CV_T	0.0		
		LAT_{INI} (ms)	NA		
		LAT_{END} (ms)	NA		
14	LSW	Nº ELE	19007	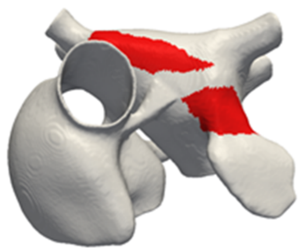	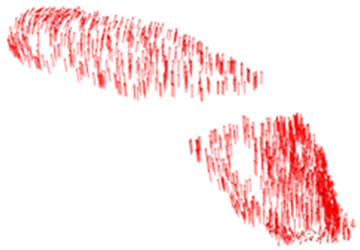
		Sub-regions	2		
		V (mm³)	513.2		
		MODEL	LA		
		MATERIAL	LA		
		CV_L	63.3		
		CV_T	36.6		
		LAT_{INI} (ms)	38		
		LAT_{END} (ms)	81		
15	LAS	Nº ELE	37094	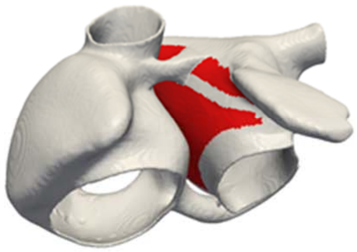	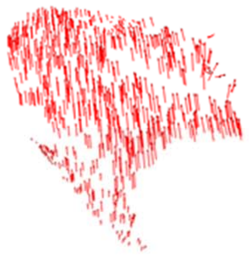
		Sub-regions	–		
		V (mm³)	1001.5		
		MODEL	LA		
		MATERIAL	LA		
		CV_L	63.3		
		CV_T	36.6		
		LAT_{INI} (ms)	40		
		LAT_{END} (ms)	83		
16	LAA	Nº ELE	31711	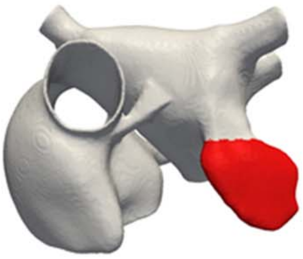	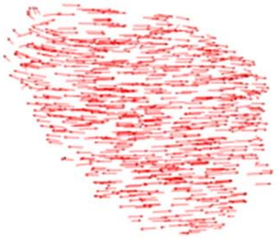
		Sub-regions	2		
		V (mm³)	856.2		
		MODEL	LAA		
		MATERIAL	LA		
		CV_L	63.3		
		CV_T	36.5		
		LAT_{INI} (ms)	64		
		LAT_{END} (ms)	125		

Nº	REGION	PROPERTY	VALUE	FIBRE DIRECTION	
17	LPW	Nº ELE	61325	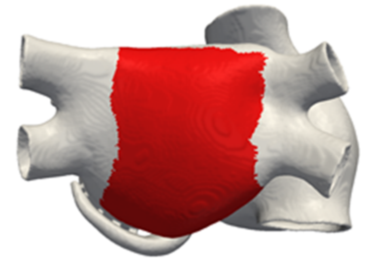	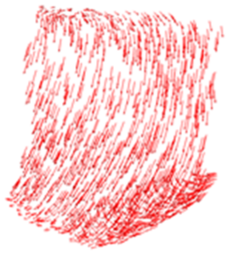
		Sub-regions	–		
		V (mm^3)	1655.8		
		MODEL	LA		
		MATERIAL	LA		
		CV_L	63.3		
		CV_T	36.6		
		$LAT_{INI\ (ms)}$	64		
		$LAT_{END\ (ms)}$	128		
18	MV	Nº ELE	21476	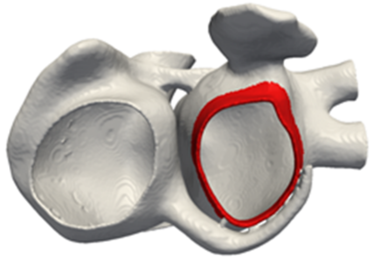	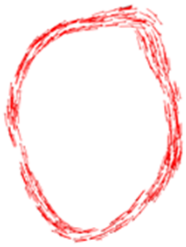
		Sub-regions	2		
		V (mm^3)	579.9		
		MODEL	MV		
		MATERIAL	LA		
		CV_L	62.9		
		CV_T	36.5		
		$LAT_{INI\ (ms)}$	64		
		$LAT_{END\ (ms)}$	116		
19	RPV	Nº ELE	30588	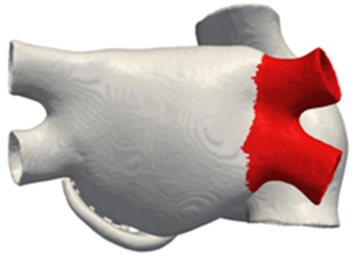	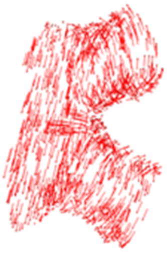
		Sub-regions	6		
		V (mm^3)	825.9		
		MODEL	PV		
		MATERIAL	PV		
		CV_L	75.0		
		CV_T	53.0		
		$LAT_{INI\ (ms)}$	64		
		$LAT_{END\ (ms)}$	91		
20	LPV	Nº ELE	45026	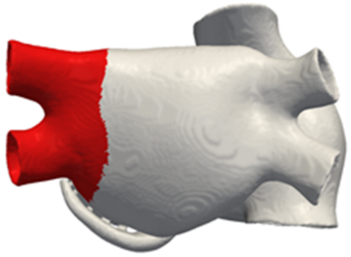	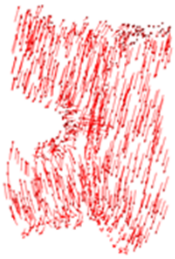
		Sub-regions	6		
		V (mm^3)	1215.7		
		MODEL	PV		
		MATERIAL	PV		
		CV_L	75.0		
		CV_T	53.0		
		$LAT_{INI\ (ms)}$	67		
		$LAT_{END\ (ms)}$	108		
21	CS	Nº ELE	21873	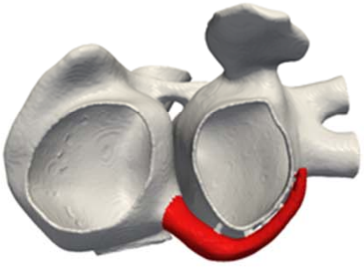	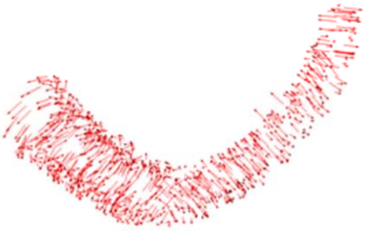
		Sub-regions	3		
		V (mm^3)	590.6		
		MODEL	RA		
		MATERIAL	CS		
		CV_L	97.2		
		CV_T	68.6		
		$LAT_{INI\ (ms)}$	75		
		$LAT_{END\ (ms)}$	132		
	CS (Unions)	Nº ELE	611	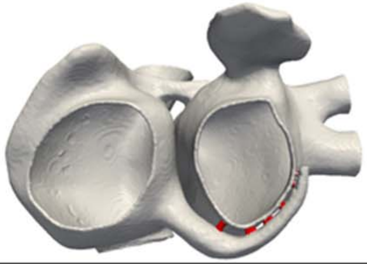	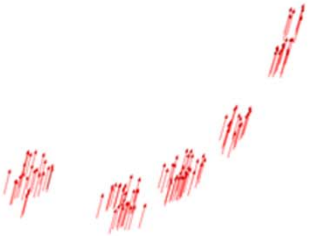
		Sub-regions	1		
		V (mm^3)	16.5		
		MODEL	RA		
		MATERIAL	LA		
		CV_L	63.3		
		CV_T	36.6		
		$LAT_{INI\ (ms)}$	75		
		$LAT_{END\ (ms)}$	132		
